# Inference and dynamic simulation of malaria using a simple climate-driven entomological model of malaria transmission

**DOI:** 10.1371/journal.pcbi.1010161

**Published:** 2022-06-09

**Authors:** Israel Ukawuba, Jeffrey Shaman

**Affiliations:** Columbia University, Mailman School of Public Health, New York, New York, United States of America; National Institutes of Health, UNITED STATES

## Abstract

Given the crucial role of climate in malaria transmission, many mechanistic models of malaria represent vector biology and the parasite lifecycle as functions of climate variables in order to accurately capture malaria transmission dynamics. Lower dimension mechanistic models that utilize implicit vector dynamics have relied on indirect climate modulation of transmission processes, which compromises investigation of the ecological role played by climate in malaria transmission. In this study, we develop an implicit process-based malaria model with direct climate-mediated modulation of transmission pressure borne through the Entomological Inoculation Rate (EIR). The EIR, a measure of the number of infectious bites per person per unit time, includes the effects of vector dynamics, resulting from mosquito development, survivorship, feeding activity and parasite development, all of which are moderated by climate. We combine this EIR-model framework, which is driven by rainfall and temperature, with Bayesian inference methods, and evaluate the model’s ability to simulate local transmission across 42 regions in Rwanda over four years. Our findings indicate that the biologically-motivated, EIR-model framework is capable of accurately simulating seasonal malaria dynamics and capturing of some of the inter-annual variation in malaria incidence. However, the model unsurprisingly failed to reproduce large declines in malaria transmission during 2018 and 2019 due to elevated anti-malaria measures, which were not accounted for in the model structure. The climate-driven transmission model also captured regional variation in malaria incidence across Rwanda’s diverse climate, while identifying key entomological and epidemiological parameters important to seasonal malaria dynamics. In general, this new model construct advances the capabilities of implicitly-forced lower dimension dynamical malaria models by leveraging climate drivers of malaria ecology and transmission.

## Author summary

Climate plays a fundamental and complex role in malaria transmission, by acting on multiple aspects of mosquito ecology and parasite transmissibility. However, to express malaria transmission pressure, malaria models with implicit vector dynamics have relied on indirect predictors of vector ecology, such as temporal seasonality or interpolations of rainfall/temperature, instead of entomological processes directly informed by ambient conditions. This approach obscures the specific influence of environmental conditions on relevant vector and parasite ecology, as well as meaningful interpretation of climate variability within these models. Here, we demonstrate that both interpretability and ecological effect from climate can be instantiated in lower dimension dynamical models through representation of transmission pressures via a climate-driven Entomological Inoculation Rate (EIR). This process-based model framework is driven by local rainfall and temperature, which regulate multiple aspects of the EIR, namely mosquito density, host-seeking activity, and parasite infectivity. Our results indicate that the climate-driven model construct is able to reproduce regional and local malaria transmission at seasonal and inter-annual time scales, while enabling identification of key entomological determinants of transmission.

## Introduction

Every year, malaria infects more than 200 million people and causes about 400,000 deaths [1]. More than 90% of this burden of disease is borne by individuals in sub-Saharan Africa, most of whom are children and pregnant women [1]. Global efforts to combat the disease have led to its elimination in several regions around the globe [2–4]. Across Africa, scale-up of interventions, beginning with the Roll Back Malaria Initiative launched by WHO in the late 1990s, has supported declines in rates of malaria deaths [1,5,6]. Understanding the local drivers of transmission and the conditions promoting exposure to the malaria vector are important for effectively controlling the spread of malaria.

The malaria parasite is transmitted to individuals by infected female *Anopheles* mosquitoes. The population dynamics of these vectors, like most ectothermic insects, are moderated by ambient conditions such as rainfall and temperature [7,8]. Additionally, temperature influences transmissibility of the malaria parasite [9,10]. Recognition of these effects of climate on the vector and parasite has led to the formulation of explicit and implicit mechanistic climate-driven models that simulate malaria and are used to elucidate the effects of local conditions on mosquito populations and malaria transmission. In explicit formulations, states of transmission in host and vector populations, as well as progression through stages of the mosquito lifecycle, are modelled in time and space. As a result, the force of infection is a direct result of the interactions between host and vector populations as the transmission system evolves in time. In implicit approaches, however, transmission in the host community is the primary focus. Instead of following transmission and life stages in vector populations, a parsimonious representation of mosquito dynamics is used to simulate the force of infection acting on the host population. The force of infection, consequently, is a result of the interaction between explicitly simulated host infection dynamics and the implicitly represented mosquito dynamics.

Both explicit [11–16] and implicit [17–20] climate-based mechanistic forms are capable of simulating malaria transmission dynamics; however, current implicit models have relied on indirect climate modulation of the force of infection [17–20]. In these implicit forms, the use of strongly correlated predictors of vector dynamics, such as observations of mosquito density or temporal seasonality and smoothing interpolations of climate [17,20,21] compromises meaningful investigation of the influence of climate on mosquito ecology. In contrast, in explicitly forced climate-driven mathematical malaria models, climate directly regulates mosquito ecology and parasite transmissibility. Implicit models provide a simple yet powerful form for capturing the seasonal and spatial spread of mosquito-borne diseases; however, the absence of a biologically-motivated climate-driven modulation of malaria transmission obscures meaningful interpretation of the specific role of climate on disease dynamics. Therefore, there is a need to enhance the implicit model forms by providing biological grounding for the effects of climate.

In this study, we show that an implicit process-based model with direct climate modulation of the force of infection through the malaria Entomological Inoculation Rate (EIR) can relatively accurately simulate observed incidence in Rwanda–a malaria endemic country. The EIR, defined as the number of infective bites received per day per human [22], depicts the influence of mosquito population dynamics and infection in the host population using a compact measure of the transmission pressure experienced by a host population. Multiple components of the EIR (namely mosquito density, mosquito feeding activity, longevity and parasite inoculation) are regulated by temperature and rainfall conditions. These various modulatory roles of ambient climate conditions on the malaria mosquito and parasite enable derivation of a climate-driven transmission pressure through the EIR.

The EIR has been used within a number of mathematical model constructs to convey the intensity of transmission due to mosquito dynamics. For example, Killeen et al. developed a deterministic model that calculates the EIR experienced by human populations by acknowledging changes in life histories of mosquitoes and allowing average temperature conditions to influence parasite transmissibility [23]. Similarly, Eckhoff [24] formulated a cohort/individual mosquito population model with explicit representation of larval development, mortality and parasite sporogony (i.e. the development of malaria parasites into sporozoites–the stage infective to hosts), which are impacted by weather conditions. A large-scale individual-based human transmission model then combines with the vector model to predict EIR. Other studies have used climate-driven explicit mosquito and human population models to simulate EIR values as crude estimates of malaria activity and for validation against field estimates [16,25].

Here, we use the EIR to represent the force of infection and allow the EIR to be directly modulated by climate through established empirical relationships between climate and the malaria vector and parasite. Similar expansions of other malaria indicator variables, include vectorial capacity (VC) and the reproductive number (R_0_), in connection with climate regulation [26,27]; however, our incorporation of the direct influence of climate on EIR in a simple, implicitly-forced dynamical model of malaria is novel. Model parameters for temperature-regulation of vector and parasite biology were based on literature, whereas the parameters of rainfall-modulation were estimated based on data from field studies and fitting of the malaria transmission model. To infer model parameters and simulate malaria incidence, the EIR-based model is paired with a data assimilation method–the Ensemble Adjustment Kalman Filter (EAKF). Similar model-inference systems using the EAKF have been developed for other infectious disease systems, have demonstrated good fit to data, and have been used to estimate critical epidemiological and entomological parameters [28–31]. Incidence and EIR levels predicted by the model-EAKF system are subsequently compared with observed malaria outcomes for local settings in Rwanda. In addition, model inferred parameter estimates of malaria transmission are compared with existing estimates to assess the validity of the model-EAKF system.

## Results

### Model simulation

**Fig 1:**
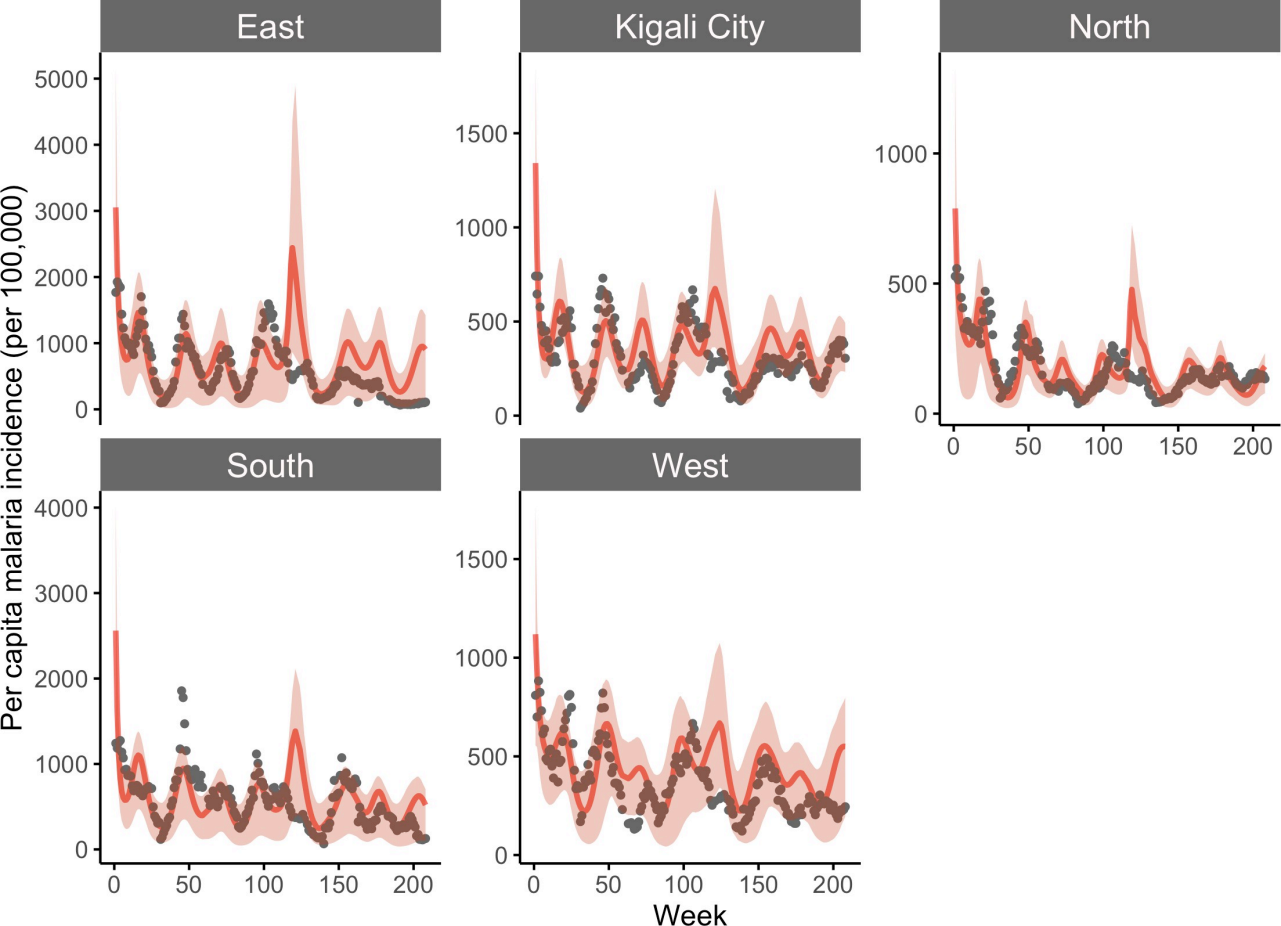
Model-EAKF simulated incidence of malaria at the province level from 2016–2019. The aggregated results (per capita incidence) from simulations of malaria transmission using the model-EAKF system. Model predictions are shown in red lines, with red shading indicating the 95% credible interval (CI) of model estimated incidence, and reported incidence is shown as gray dots. Note that the range of y-axes for model simulated and reported malaria differ among panels.

**Fig 2:**
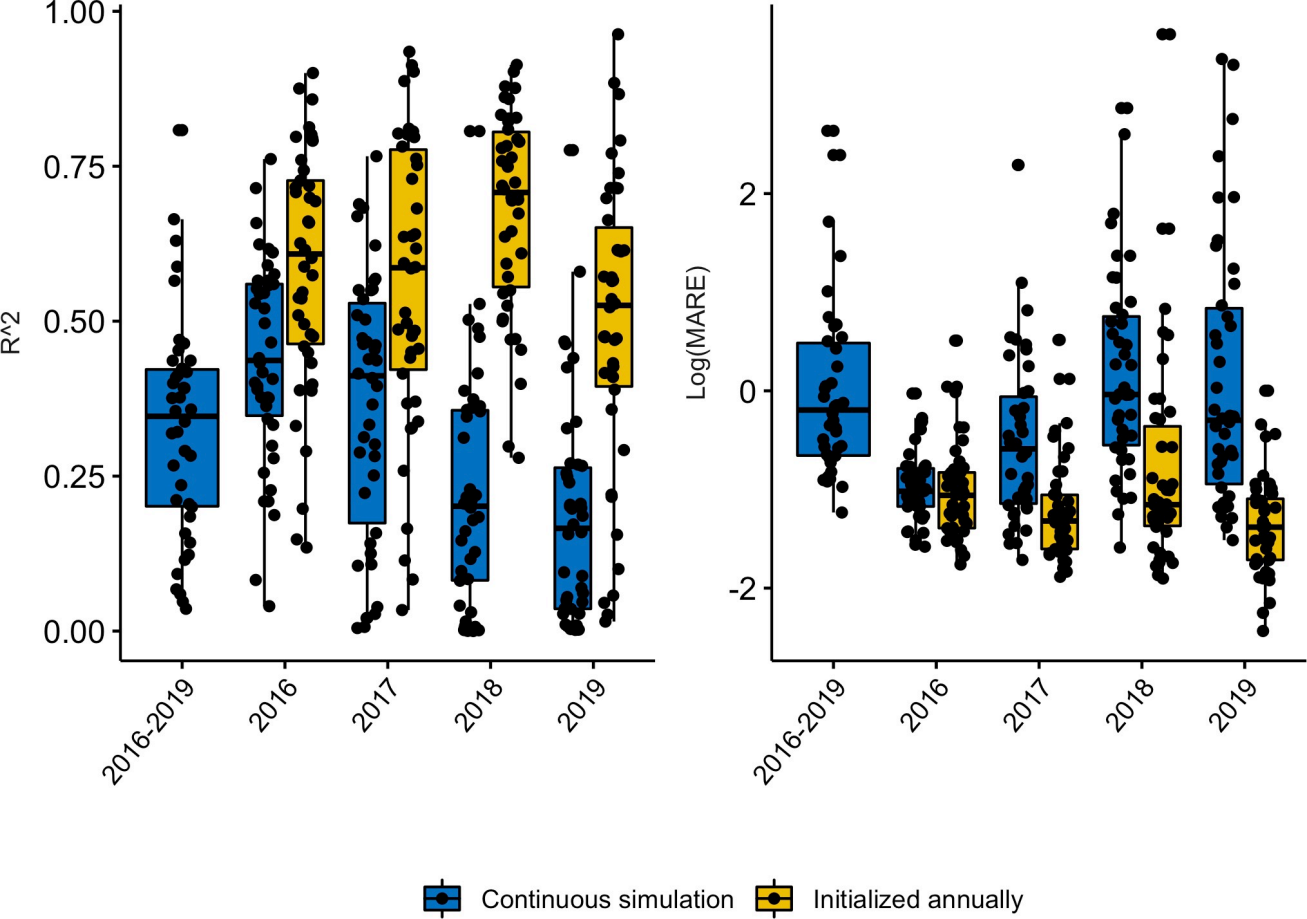
Fit of predicted malaria incidence, across all 42 catchment sites, computed over various study periods. Outcomes computed from continuous weekly simulation of incidence are indicated by the blue boxes. Outcomes computed from yearly reinitialization are indicated by the yellow boxes. Left panel: the coefficient of determination (R^2^). Right panel: log-scaled Mean Absolute Relative Error (MARE) across different sites (jittered black dots).

**Fig 3:**
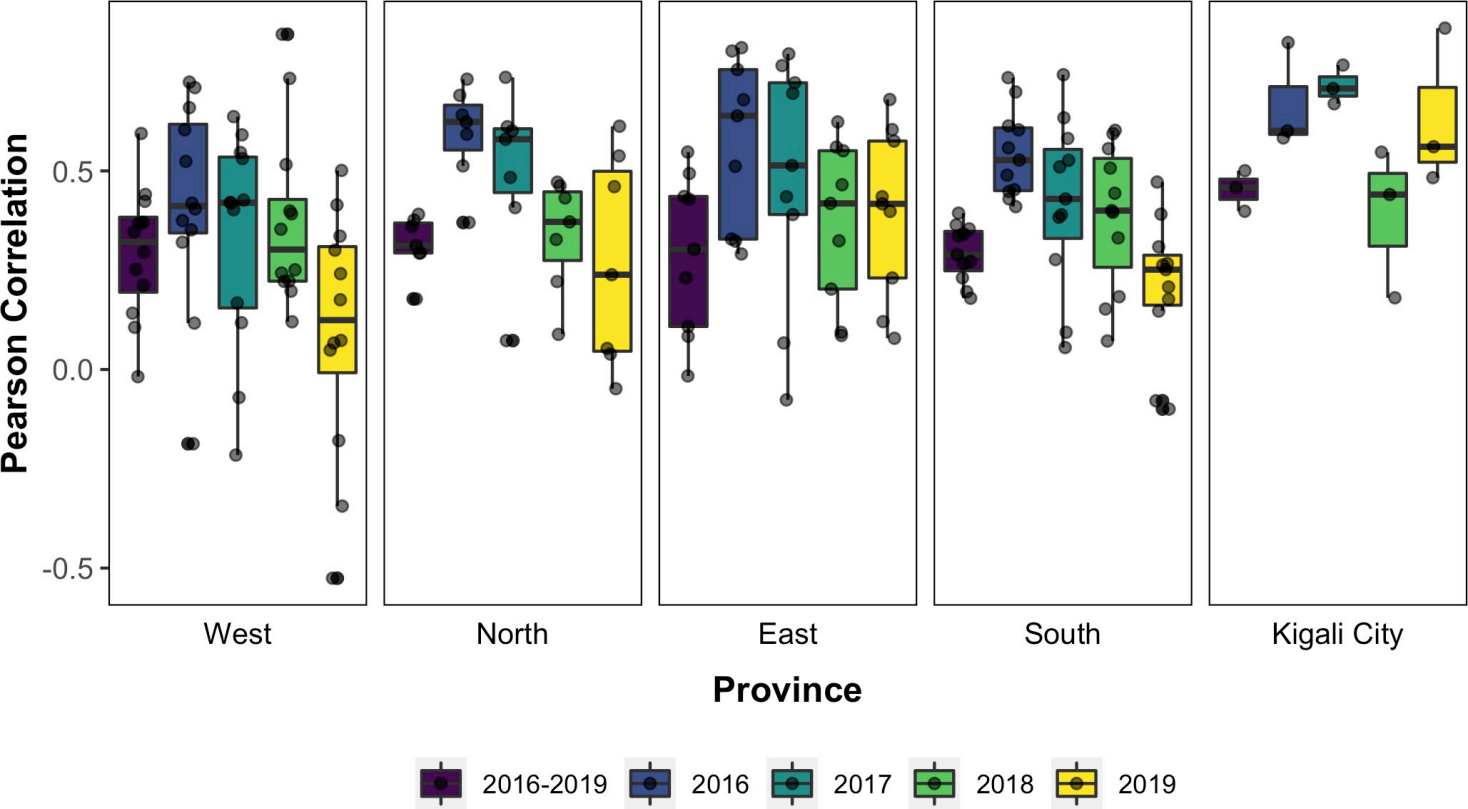
Correlation between reported malaria incidence and cumulative moisture conditions for the final model. The Pearson Correlation coefficients estimated for study sites (jittered black dots) over the entire study period are in purple and estimates for each individual year (i.e. 2016 to 2019) are in in blue, green, light green, and yellow, respectively.

Fitting of the dynamical model occurred at the more resolved local catchment level, while province level predictions were taken as the aggregate of local simulations. Results from model fitting show that the dynamical model captures the seasonal dynamics and some of the inter-annual variability observed in malaria incidence at aggregated province levels (Fig 1) and public health catchment levels (supplemental Fig A in S1 Text). With simulated incidence aggregated to the province level, as shown in Fig 1, the model-EAKF system explained 27.75–65.64% of malaria variability; at the finer catchment level, it captured 3.6%– 80.82% of variability observed in malaria incidence in all sites from 2016–2019. Performance of the model-EAKF system was generally higher earlier in the study period, with a median of 43.66% and 41.19% of incidence variance explained across all sites in 2016 and 2017, respectively (Fig 2, blue boxplots). Model error was also lower during these seasons. Subsequent years saw diminished model fit, with a median variance of 20.47% and 16.58% simulated by the model-inference system during 2018 and 2019. During these seasons, the model-EAKF system predicted more intense transmission in 2018, followed by less intense transmission during the 2019 season. In contrast, large declines of malaria incidence occurred, including a complete disruption of malaria seasonality. This disconnection between the climate-driven model and malaria incidence is further highlighted by the progressively dwindling association between malaria incidence and rainfall conditions within the model for most of Rwanda (Fig 3). Nonetheless, the role of rainfall is evident in the seasonality of malaria incidence. The bimodal peaks and troughs in predicted malaria incidence appear to be connected to the two distinct–long (March to May) and short (October to December)–rainy seasons in Rwanda.

**Fig 4:**
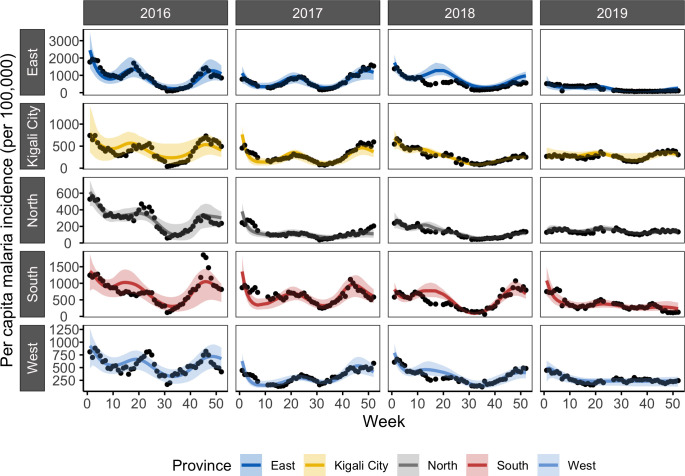
Incidence of malaria as simulated by the model-EAKF system under yearly initialization. The aggregated results (per capita incidence) from simulations of malaria transmission using the model-EAKF system, while re-initializing each year separately. On the y-axis of each panel, corresponding to different provinces, reported incidence is shown as black dots, while model-EAKF fittings are shown in colored lines. Colored shading indicates the spread of the 95% CI of model estimated incidence. Aggregated simulations for sites in the East, Kigali City, North, South and West provinces are indicated in blue, yellow, grey, red and light blue, respectively.

Because of the large disruption in the year-to-year cycle of malaria transmission, we investigated whether the model-EAKF system could better constrain intra-annual malaria activity by re-initializing the system each year (Fig 4). By fitting and simulating each year separately, malaria incidence variability was better captured at the province level, with 41.18%–86.89% of variance explained. At the more resolved catchment level, median variance explained rose as well– 60.84%, 58.62%, 70.77% and 52.55% in 2016–2019 respectively (Fig 2, yellow boxplots). This exercise was conducted annually in order to countercheck the capacity of the model-EAKF approach to capture malaria activity in the later study period, which was poorly represented under continuous simulation. Although re-initializing each year improves the model simulations, we caution that it could lead to over-fitting and overly confident explanation of the data, particularly when plausible external factors affecting transmission (e.g., intensified control) are unaccounted. For this reason, we have based our conclusions and final reports of incidence, EIR and other malaria parameters on findings obtained under the continuous simulation with the model-EAKF system.

### Ento-epidemiological parameters of malaria

The average duration of *Plasmodium falciparum* incubation within the host population (μ_EI_) was inferred by the model-EAKF system to last between 9.17–13.08 days. These site-level estimates were fairly similar throughout the provinces of Rwanda (Table 1). Depending on the region and site, *Plasmodium falciparum* incubation (μ_EI_) tended to be longer or shorter by no more than a day. However, estimates of the duration of subpatency for individuals recovering from untreated infections (μ_RS_) were less similar among sites (Fig C in S1 Text). Study sites in the East, Kigali City, North, South, and West provinces were found to have median subpatency durations of 265.59 days, 240.08 days, 231.21 days, 246.13 days and 274.85 days, respectively (Table 1 and Table A in S1 Text). The relative infectivity of subpatent infections compared to clinical infections (q_R_) was estimated to be within similar ranges (0.05–0.13) for the majority of the sites (Fig C in S1 Text; Table A in S1 Text). Additionally, malaria transmission parameters were in generally comparable ranges whether model inference was conducted continuously during the study period or initialized separately each year (Fig D in S1 Text).

**Table 1:** Mean posterior estimates for two epidemiological parameters of malaria. μ_EI_ is the average duration of parasite incubation in humans and μ_RS_, the average duration of subpatent infection. The province median and range of the mean estimate for sites within a province are shown for regional comparison.

Province	Posterior estimate (days) for Study Sites	
–	μ_EI_ median (range)	μ_RS_ median (range)
East (N = 9)	10.58 (10.39–13.08)	265.59 (140.74–345.08)
Kigali City (N = 3)	11.22 (11.14–11.67)	240.08 (238.85–281.20)
North (N = 7)	11.05 (9.65–12.08)	231.21 (124.22–344.36)
South (N = 11)	10.68 (9.17–12.72)	246.13 (151.62–348.75)
West (N = 12)	10.64 (9.56–12.26)	274.85 (145.01–355.16)

N = number of sites located within a province.

**Fig 5:**
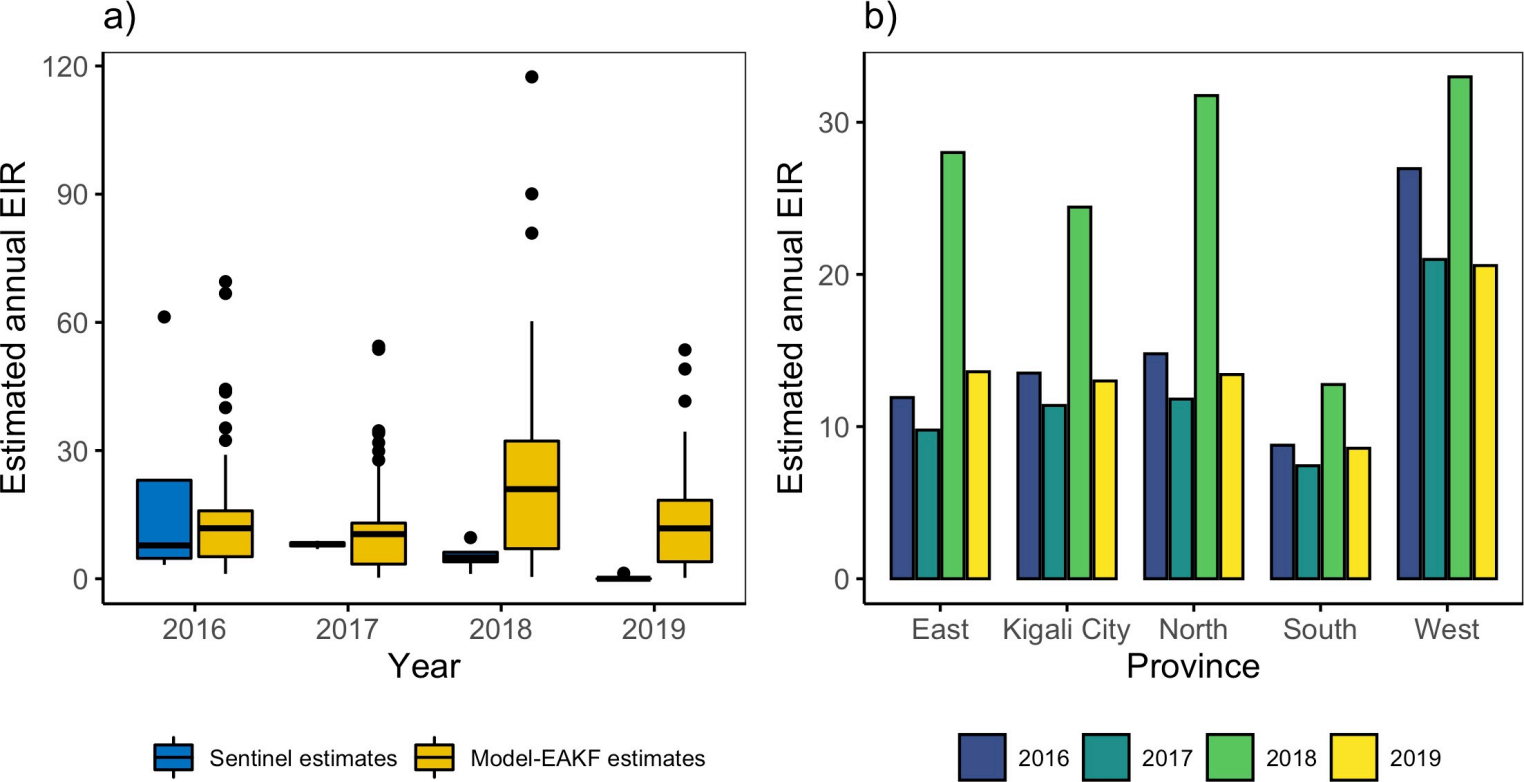
Estimates of the annual Entomological Inoculation Rate (EIR) across Rwanda 2016–2019. (a) Side-by-side boxplots of field estimated annual EIR from four entomology sentinel sites [32–35] across Rwanda (blue boxplots) and of model estimates of annual EIR for 42 study sites generated by continuous simulation of the model-EAKF system (yellow boxplots) for 2016–2019. (b) Average annual EIR at the province level aggregated from continuous simulation of the model-EAKF system at the site level. Blue, green, light green, and yellow colored bars represent 2016, 2017, 2018 and 2019, respectively.

A relatively stable entomological exposure, measured by the annual EIR, was estimated among sites in Rwanda during most of the study period. However, the 2018 transmission season was an exception and experienced a predicted increase in EIR exposure throughout Rwanda (Fig 5). This was in accordance with the intense transmission predicted for 2018 due to heightened environmental capacity and suitability for mosquito survivorship and development (see supplement, Fig B in S1 Text). During the other, stable years, individuals were estimated to experience an annual EIR of 0.19–69.53 infective bites per person per year (bpy) (Fig 5A). However, in 2018, individuals were estimated to receive about 1.80 times more infective bites per person. By region, estimated entomological exposure varied moderately across Rwanda. Populations in the North and West were predicted to experience up to 1.79–2.70 times more infective bites per person per year than other regions (Fig 5B). The high levels of entomological exposure reflect the high attack rates of sites for the West; however, when the relatively smaller effective denominator population for these areas (Fig J in S1 Text) are accounted, the predicted force of infection increases and corroborates estimated EIR (Fig K in S1 Text). Furthermore, these communities were predicted to have higher mosquito-survivorship due to more suitable surface moisture conditions than the rest of the country (Fig B in S1 Text).

Malaria transmission within the climate-driven model is regulated through a number of pathways. Analyses indicate that malaria dynamics were most affected by rainfall regulation of sub-adult survivorship. Without rainfall-modulated sub-adult development, model fitness decreased across sites, with MARE climbing by more than 40% in the final model (Fig E in S1 Text). Temperature-regulated conditions such as parasite sporogony (n), biting rate (a) and development rate (P_EAT_) contributed to model fitness but played a far less substantive role constraining the model fitting. The absence of these temperature-forced pathways only slightly decreased model fitness by about 3% maximum.

## Discussion

Temperature and rainfall are major drivers of malaria vector population dynamics and parasite transmission. The effects of these meteorological conditions on the ecological life traits of the malaria vector and parasite make them important for accurately describing transmission risk. Several malaria modeling studies have demonstrated that malaria and other mosquito-borne diseases indeed can be accurately described using an implicit force of infection that depends on indirect climate predictors of mosquito dynamics [17,20,21]. However, our study finds that by leveraging the direct ecological effect of climate on EIR, accurate simulation of malaria dynamics can be achieved using an implicitly-forced dynamical model of malaria transmission. The model structure accurately reproduced seasonal malaria incidence and some of the inter-annual variability, without need for explicit representation of the vector population and transmission dynamics. But, inter-annual malaria activity in some years was less accurately captured, likely due to increased malaria control that was not included in the model. The developed EIR-model framework also captured geographic differences in malaria incidence across the varied climate regimes of Rwanda and enabled formal investigation of the role of climate in mediating the risk of malaria transmission. However, areas with higher model predicted EIR did not necessarily correspond with higher malaria incidence; geographic differences in population-level immunity and entomological control are suspected to have altered the climate-predicted EIR experienced by host populations.

Over the long term, the climate-driven model reproduced malaria incidence fairly well. However, some notable disagreement, particularly in the last two years of study, between observations and model simulations was evident. This observed disconnection suggests action of external factors, not represented in the model, disrupting vectorial capacity and precluding transmission. Specifically, a nationwide increase in transmission was predicted following initial years of high agreement between climate-signaled transmission and malaria incidence. However, declines and disrupted seasonal activity were reported across Rwanda. Indeed, starting in 2018, several high malaria burden regions in Rwanda began intensified malaria control in an effort to recover gains toward malaria elimination lost due to relaxed malaria control [36–38]. This national malaria control program in Rwanda largely relies on long-lasting insecticide treated bednets (ITNs) and indoor residual spraying (IRS) [39], which are highly effective measures for reducing entomological exposure risk. ITN and IRS not only lower mosquito density but also reduce mosquito-host contact rates as well as mosquito oviposition, doubly lowering malaria vectorial capacity [40–42]. Additionally, their long-lasting mode of action ensures a continuous effect on the risk of entomological exposure throughout the transmission season. Thus, the re-adoption and upscaling of these blanket IRS and ITN campaigns, particularly in regions in the East and South provinces where malaria is more intense, may explain the drastic depression of seasonal malaria incidence and low entomological inoculation rates reported in the last two study years across Rwanda [37,38].

In addition to capturing local dynamics, the climate-driven model also identified parameters best describing local malaria that are similar to previous malaria estimates, lending more external validity to the model. While malaria control in Rwanda increased during the latter portion of the study record, the parameter estimates remained relatively stable from year to year. Widespread control measures sustained over long periods of time have been known to induce changes in population epidemiology such as population-level immunity [43,44], as transmission become effectively interrupted and eliminated. However, given the short timescale of control observed here, as well as the prevalence of active transmission in Rwanda, key parameters such as immunity might not have drifted substantially.

Model-EAKF inferred estimates for Rwanda are also in agreement with those made more broadly for *Plasmodium falciparum*, which dominates transmission in Rwanda, like in all of sub-Saharan Africa [1]. *Plasmodium falciparum* infections are characterized by short incubation periods before symptom onset as well as relatively short periods of patency for untreated infections compared to other malaria species. Our model-inferred duration of *P*. *falciparum* incubation and relative infectivity of subpatent infections agree with previous epidemiological estimates [45–48] and only varied slightly across Rwanda; however, malaria patency estimates were less uniform. This variation in susceptibility could be a result of differences in malaria burden, which may complicate acquired immunity across populations [49–51].

The intensity of malaria transmission is not uniform across Rwanda, but varies from region to region [36,52]. The level of parasite circulation within a community alters the rate of repeated exposure to malaria parasites and may lead to differential rates of susceptibility and acquired immunity observed within a population [43,50]. Additional population factors at play include community age structure, level of general health, and migration [49–51], which may preclude a uniform rate of susceptibility and acquisition of malaria immunity across study populations.

The malaria model framework also allowed approximation of the EIR–an indicator of the intensity of transmission. Model predicted levels of annual EIR (0.19–114.45 bpy) were low compared to levels in other neighboring malaria endemic countries [53], but higher than found using empirical relationships (0.48 bpy) and dynamic simulation (2.2 bpy) in 2010 at the country-level [54]. A comprehensive entomological field study from 2010–2013 [55], comprised of 12 malaria sentinel sites spanning the country, however found annual EIR ranging from 0.99–329.01 bpy throughout Rwanda. More recent surveys from 2016–2019 similarly suggest, that although malaria exposure risks have dropped in Rwanda, mostly due to intensified control measures, field estimates of annual EIR rates remain higher (0–61.270 bpy) than those earlier model estimates [32–35]. The model-EAKF system estimates presented here for the early portion of the study period closely agree with these recent field estimates. Differences between model and field estimates could be a result of vector and parasite control activities, which are currently uncaptured in the model. The ramping up of intensified control measures by national malaria control programs in Rwanda, during the later years of the study [36–38], might explain the steady decline of entomological exposure risks away from the high EIR levels estimated by our model.

Across the diverse mountainous geography of Rwanda, a non-uniform rate of entomological exposure was found among the five provinces. In the lowlands, mostly situated in the South and East provinces, climate suitability for malaria transmission can be expected to be higher than in the highlands in the West and North because of generally warmer conditions that are closer to the optimal temperature for malaria transmission. In contrast, our model estimates suggest higher EIR levels in the less warm but wetter West and North regions, and lower levels in the East and South, where rates of malaria incidence are typically higher [36,56,57]. This counterintuitive finding might result from differences in malaria care seeking. Increased population-level immunity due to a higher malaria burden in the South and East could manifest as milder malaria episodes [51,58], differentially impacting treatment seeking behavior. As a consequence, a lower entomological exposure could be inaccurately inferred for the region.

In addition, the high EIR estimates in the West may partly be explained by more focal transmission, suggested by the smaller effective transmission population size estimated for this region (see population scaling parameter [s] in Methods). Indeed, conditions are relatively cooler in these regions and could limit the geographical range of malaria activity relative to low altitude regions [9,59,60]. The lower denominator population from the pockets of sub-communities with more climatically suitable environments combined with comparatively low population-level immunity [58,60–62] could result in higher EIR rates, while much of the high-altitude populations could remain generally less involved due to low parasite suitability. When ‘at-risk’ denominator population is accounted for, predicted malaria rates suggest higher risk per capita in historically low-burden areas compared to historically high-burden regions, in a pattern that corresponds with the predicted pattern of EIR exposure (see Figs J and K in S1 Text).

An important benefit of direct climate-mediation of transmission pressures is the ability to examine the relative contribution of climate-driven vector ecology on malaria risk. Ecological pathways to transmission could reveal insights for better exploiting the malaria vector dependency on environmental conditions. In this study, rainfall-regulated sub-adult survivorship proved to be the entomological factor most impactful to local transmission, well above the temperature-regulated vector and parasite dynamics represented in the transmission system. Note that temperature acting on adult mortality was not assessed due to the lack of temperature-regulated adult mortality in our model (see Methods). Thus, our system may underestimate the role of temperature, as adult mortality is recognized as a major driver of malaria vectorial capacity [63–65]. However, among the temperature-dependent ecology evaluated, seasonal effects of parasite transmissibility, sub-adult duration and survivorship or feeding activity were slight and lead to negligible impacts on the seasonal dynamics of malaria transmission. The role of rainfall in malaria vectorial capacity in the region is highlighted further by the distinct dynamics of malaria seasonality, which correspond with the two rainy seasons. This finding is in agreement with several other studies [66–70] that have linked rainfall and soil moisture conditions in East and sub-Saharan to *Anopheles* mosquito activity and malaria transmission more than to temperature. The environmental carrying capacity for malaria mosquitoes (i.e., the population size that resources in the environment can support) is thought to be defined by moisture conditions [71,72]. *Anopheles* mosquitoes begin their life in water and continue developing in this aquatic environment until adult emergence. The formation and stability of these rain-fed, short-lived water pools are likely to be more impacted by rainfall variability than temperature, which is near optimal levels for mosquito development throughout the year.

This study demonstrates that accurate description of malaria over seasonal and inter-annual time scales can be achieved by implicitly forced parsimonious process-based malaria models while elaborating the direct role of climate on parasite and mosquito ecology. The use of direct climate modulation of vector ecology, as shown here, further extends the utility of malaria transmission models with implicit vector dynamics for examining climate drivers of local transmission. Future modelling studies will evaluate the benefit of using similar climate-forced models for forecasting local malaria incidence and will also assess the predictive utility of the entomological model framework in supporting ongoing local malaria control efforts in sub-Saharan Africa.

## Methods

### Model description

#### Compartmental model

**Fig 6:**
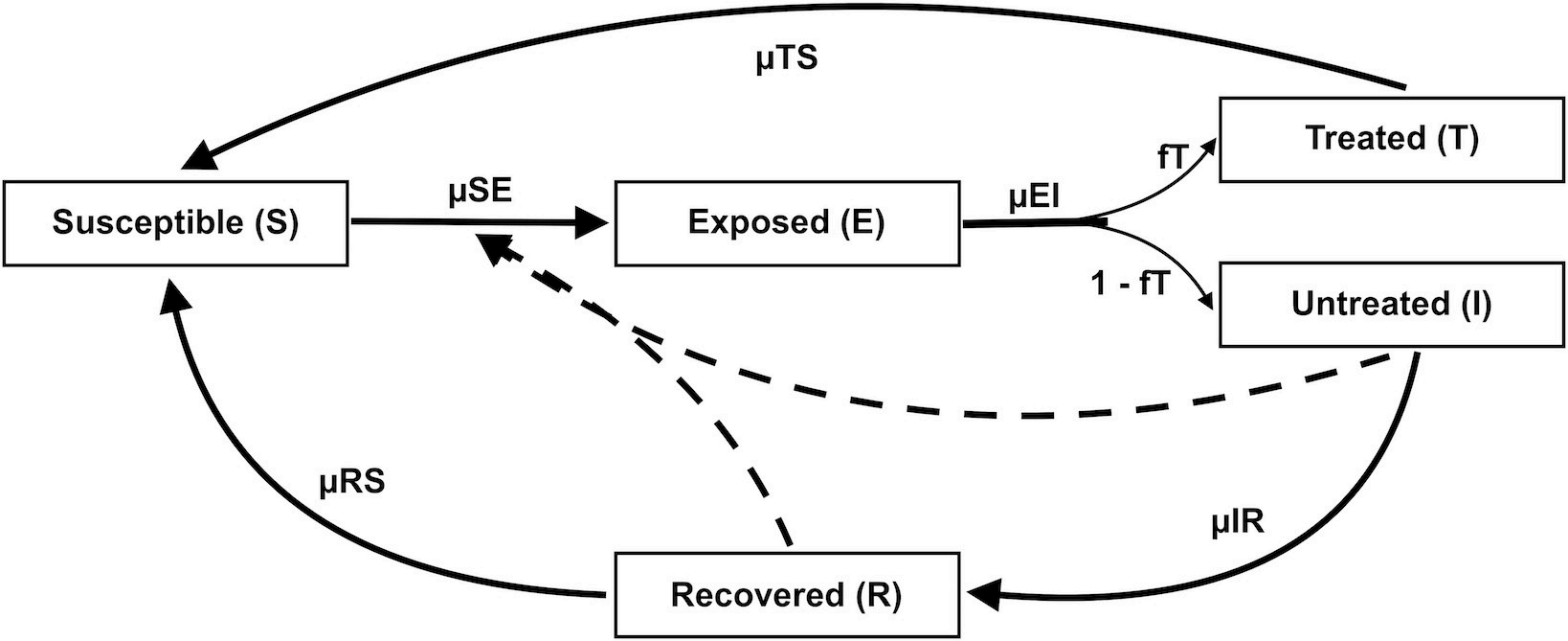
Flow diagram of the malaria transmission model. Individuals within the transmission system are divided into those who are susceptible to infection (S), exposed to the parasite (E), have clinical infection and are treated (T), have clinical infection and are untreated (I), and have recovered from clinical disease (R). Individuals are susceptible at birth (μ_BS_) or following loss of immunity (end of patency) or end of treatment–related prophylaxis. Susceptible individuals become exposed at a rate determined by the force of infection (μ_SE_). Exposed hosts (E) then undergo intrinsic incubation of *Plasmodium falciparum* at a rate determined by μ_EI_. Following parasite incubation, infected cases begin to show clinical symptoms, a fraction (f_T_) of which are assumed to be fully treated (state T) with the remainder untreated (state I). Treated individuals return to the susceptible state at a rate determined by the duration of the prophylactic effect (μ_TS_) due to antimalarial therapy. Untreated infections recover naturally (μ_IR_) from clinical disease and show no further symptoms of clinical infection. Hosts that naturally recover, enter a state of low level parasitemia (state R) that is tolerated by the immune system. Under this patent/subpatent state, individuals are protected from reinfection and have a relatively lower infectivity to mosquitoes (q_R_) compared to full-blown clinical infections. Once patent/subpatent infections are cleared (μ_RS_), recovered hosts again become susceptible.

To represent malaria transmission, we developed a compartmental model with a Susceptible Exposed Infected Recovered and Susceptible (SEIRS) form, with an additional state T for treated individuals (Eq 1–5). Within this system (Fig 6), the human population is divided among individuals who are susceptible to infection (S), exposed to the parasite but not yet infectious (E), have clinical infection and are treated (T), have clinical infection and are untreated (I), and recovered from clinical disease (R). Hosts transition between susceptible, exposed, untreated, treated and recovered states at rates determined by the model parameters, which represent key epidemiological and human demographic characteristics. Based on birth rate estimates (μ_BS_) from recent census data, individuals enter the Susceptible (S) group; previously infected individuals can also return to the susceptible state due to loss of immunity (end of patency) or the end of treatment–related prophylaxis. The immunity structure employed here is a simplification of more complex dynamics, which are age and parasite specific and vary with repeated exposure. To maintain model parsimony, we adopt a simple structure in which previous infection confers non-sterilizing immunity in which recovered individuals become susceptible again. Similar simplified representations of malaria immunity have provided reasonable fittings of observed malaria incidence [20,21,73].

Following exposure to an infectious mosquito bite, susceptible individuals move into the exposed group at a certain rate of infection, determined by the force of infection (μ_SE_). While in the exposed state, individuals undergo intrinsic incubation of *Plasmodium falciparum* during which parasites transition to gametocytes–the parasitic stage infectious to mosquitoes. Following this incubation period (μ_EI_), which typically lasts 8–21 days in humans [45], individuals begin to show clinical symptoms, and are caught by the surveillance system. Malaria incidence in the transmission system is tracked by μ_EI_*E, the number of newly infected individuals per unit time. These clinical infections may be fully treated (state T) by anti-malaria therapy drug or remain untreated (state I). Based on recent Demographic and Health Survey (DHS) data on treatment seeking behavior of individuals with fever in Rwanda [74], half of newly occurring infections (f_T_) are assumed to seek and receive full treatment.

Treated individuals (in state T) return to being susceptible after the duration of the post-treatment prophylactic effect (μ_TS_) provided by antimalarial therapy. Only untreated infections contribute to the force of infection and can transmit the parasite to mosquitoes with a certain probability (P_HM_), as not all blood meals result in mosquito infection. Several factors such as human gametocyte density and antimalarial treatment may affect the likelihood of infecting a blood-feeding mosquito [75–77]. Untreated infections recover naturally (μ_IR_) from clinical disease and show no further symptoms of clinical infection. However, given that malaria infection can outlive clinical symptoms, recovered individuals enter a state of low level parasitemia (state R) that is tolerated by the immune system. During this period of patent/subpatent infection, individuals are protected from reinfection and have a relatively lower infectivity to mosquitoes (q_R_) compared to full-blown clinical infections. The host immune system steadily and eventually clears patent and sub-patent infections, resulting in individuals returning to susceptibility after a time based on the duration of patency and sub-patency period μ_RS_. Because the fatality rate from malaria parasite infection in host is relatively low and modified by age and pregnancy, which are unrepresented in the system, infected individuals experience no net increase in death rate. However, individuals in all compartments experience a background mortality rate (δ) determined by recent census data. The set of ordinary differential equations representing this malaria transmission system are as follows:

$$\frac{dS}{dt}=\mu_{BS}P-\mu_{SE}S+\mu_{RS}R+\mu_{TS}T-\delta S$$ (1)

$$\frac{dE}{dt}=\mu_{SE}S-\mu_{EI}E-\delta E$$ (2)

$$\frac{dI}{dt}=(1-f_{T})(\mu_{EI}E)-\mu_{IR}I-\delta I$$ (3)

$$\frac{dT}{dt}=f_{T}(\mu_{EI}E)-\mu_{TS}T-\delta T$$ (4)

$$\frac{dR}{dt}=\mu_{IR}I-\mu_{RS}R-\delta R$$ (5)

P=S+E+I+T+R (6)

#### Force of infection

The force of infection in the transmission system is dependent on the collective pressure exacted by the infectious populations of humans and vectors and is directly related to the EIR–the number of infectious bites received per person per unit time. Often estimated in the field as the product of the Human Biting Rate (HBR) and sporozoite rate (the proportion of infectious mosquitoes), the EIR at time *t* also can be expressed mathematically (Eq 7) using assumptions of human infectiousness and mosquito infectiousness, density, feeding activity and survivorship [22]:

$$EIR\;(t)=\frac{ma^{2}P_{HM}\;X\;e^{-gn}}{g+aP_{HM}X}$$ (7)

where *m* is mosquito density, *a* is the per-mosquito biting rate, *P*_*HM*_ the probability of transmission from human to mosquito, *g* the adult mosquito death rate, *n* sporogony duration and *X* the proportion of infectious humans at time *t*. We define $X=\frac{I+q_{R}*R}{S+E+I+T+R}$, as the fraction of human states (i.e., *I* untreated clinical infections and *R* non-clinical infections adjusted by their lower infectivity, *q*_*R*_) contributing to the force of infection.

Analyses of collected EIR data and new malaria infection indicate the existence of a nonlinear relationship with the force of infection [22,99,100] in which risk of infection saturates at high EIR. These findings show a clear deviation from the assumptions of the Ross-Macdonald model [101,102] that infectious bites are linearly proportional to the rate of new infections. Therefore, following Smith et al. [99] and others [103], we model a modified force of infection (*μ*_*SE*_) to acknowledge nonlinearity between infectious mosquito bites and the risk of new infections:

$$\mu_{SE}(t)=1-e^{-P_{MH}*EIR(t)}$$ (8)

The density of parasite in a vector, host immune status, and failure of the parasite to advance to blood stages all can affect whether an infectious bite produces infection in a human host [104]. Therefore, only a proportion of infectious bites *P*_*MH*_ (i.e., the probability of mosquito to human transmission) lead to human infection.

### Climate-regulation of vector and parasite dynamics

Many of the natural breeding sites used by female *Anopheles* mosquitoes for breeding and sub-adult development such as temporary pools, puddles and hoof prints are rain-fed surface waters. For *Anopheles gambiae*, the principal vector of malaria in sub-Saharan Africa, increased moisture levels enhance the hatching success rates of eggs and progression of mosquito larvae to the pupae stage of development [105,106]. Eggs of *An*. *gambiae* are more tolerant to dry conditions than larvae or pupae and are able to survive up to 12–15 days [105,107] but eventually die, when under prolonged dry periods. However, during periods of low and intermittent rainfall activity, the short-term tolerance of eggs to desiccation ensures maintenance of *Anopheles* populations and allows rebound in mosquito population levels following the return of the wet season [108,109]. Due to this modulation of environmental breeding capacity, rainfall is a strong regulator of the seasonal and spatial abundance of *Anopheles* mosquitoes and ultimately malaria transmission.

Temperature also modulates the malaria force of infection, by moderating mosquito population numbers and parasite transmissibility. *An*. *gambiae* mosquitoes complete the sub-adult life stages in about 5–8 days, if temperature conditions are optimal (20–22°C) [8]. Adults and pre-adult mosquitoes survive well at these optimum temperatures, too [8,9,110]. Additionally, temperature regulates the infectivity of *Anopheles* mosquitoes. Parasites within infected female *Anopheles* must complete a sporogonic cycle to develop into sporozoites, at which stage they are infectious to a human host. Warm temperatures (24–28°C) enhance this development rate, decrease the duration of parasite incubation (5-7days) [9,10], and consequently raise the probability of an infective contact. In contrast, extreme low or high temperatures (<16 or >35°C) result in delayed development of the parasite and a decrease in malaria transmission risk [27].

Temperature also modulates rates of vector-hosts contact. To develop their eggs [111], female *Anopheles* take at least one blood meal every gonotrophic cycle [112], during which proteins acquired from the meal are digested and used for egg maturation [113,114]. Fully matured eggs are oviposited, ending the gonotrophic cycle; surviving female *Anopheles* may then seek another meal. The interval between feeding and oviposition is modulated by temperature. Warmer conditions accelerate egg maturation and thereby decrease the time between bloodmeals [9,115]. The resulting higher frequency of host-seeking supports more oviposition and further inflates the malaria force of infection. The sections that follow below specify how temperature and rainfall conditions directly relate to mosquito and parasite dynamics within the EIR as described in Eq 7.

#### Mosquito density (m)

Following a modified version [7,8,27] of the population density model of Parham and Michael [116] describing a mosquito population at equilibrium, we define adult mosquito density as:

$$m=\frac{L(T,R)}{\mu_{M}(T)}$$ (9)

where L is the temperature and rainfall dependent birth rate of adult mosquitoes (defined by Eq 10) and μ_M_ is the mortality rate of adult mosquitoes. While existing mature mosquitoes must survive long enough to feed, reproduce and oviposit eggs at available breeding sites, pre-adult mosquitoes need to successfully move through egg, larvae and pupae stages in a timely manner to emerge and contribute to the naive adult population. Temperature conditions and water habitat availability can severely change the population balance by altering survival probabilities at all life stages and as well as female oviposition. Increased adult mortality and failure of pre-adult development because of suboptimal conditions jointly lower the reproductive power of the mosquito population and consequently adult density. These environmentally driven effects are reflected in the population model by setting the probability that eggs survive to adults (P_EA_), the egg-to-adult development time (τ_EA_), and the lifetime number of eggs laid by female adults (B), as functions of temperature and rainfall.


$$L(T,R)=\frac{B(T)\;P_{EA}(T,R)}{\tau_{EA}(T)}$$ (10)

We next describe each of these components and how temperature (T) and rainfall (R) simultaneously modulate the outcomes of pre-adult mosquitoes within the model.

#### Lifetime number of eggs (B)

The typical number of eggs laid by female *Anopheles* mosquitoes during oviposition may range from 50 to 290 [94–96]. Although the number of eggs laid have not been linked to ambient conditions, the frequency of oviposition, which is determined by the length of the gonotrophic cycle–the time between a blood meal and egg production–varies as a function of temperature [9]. Thus, by influencing the frequency of oviposition, temperature affects the number of eggs laid during the adult lifespan of a female *Anopheles*. Assuming a female mosquito oviposits every few days, depending on the length of the gonotrophic cycle, and experiences a constant rate of mortality (μ_M_), an exponential relationship with egg laying is expected. Given these assumptions, and following White et al. [71], the number of eggs produced over the female mosquito lifespan is:

$$B(T)=\frac{\varepsilon }{e^{GP\mu_{M}}-1}$$ (11)

$$GP(T)=\frac{1}{0.017T-0.165}$$ (12)

where ε is the number of eggs laid per gonotrophic cycle, GP is the length of the gonotrophic cycle, and μ_M_ is daily adult mortality rate.

#### Adult mosquito mortality (μ_M_)

Though temperature in general affects adult mosquito mortality [110], preliminary assessments showed limited transmission and more erroneous outcomes using temperature-dependent adult mortality data fittings [27,117]. During gonotrophy when time is spent resting, digesting a blood meal and developing eggs, mortality may be more affected by predation or by the use of endophily (i.e. resting indoors) versus exophily (i.e. resting outdoors) [118–120], confounding the relationship with temperature. Therefore, adult mosquito mortality in the mosquito population is fixed to a constant, temperature-independent rate (μ_M_).

#### Egg to adult survivorship

Mosquitoes begin their life in water as eggs and continue developing into larvae and pupae in this aquatic environment before emerging as adults. Though sub-adult mosquitoes are adapted to aquatic environments, they are highly sensitive to changes of temperature and the water level of their breeding habitat. Near the optimal 24–28°C water temperature, larval development can be completed in about 9–14 days [8]; at colder temperatures, larval duration can take 7–14 days longer. Larval mortality also increases by 30–70% [7], as temperature shifts away from optimality. Additionally, significant drops in water habitat level or prolonged periods of desiccation, rapidly decreases the probability of egg, larvae and pupae survival [105,106]. The critical roles of rainfall and temperature are readily highlighted by the spatial and seasonal dynamics of *Anopheles* mosquitoes. Following the onset peak of the rainy season, vector abundance steadily rises and peaks [108,109], as frequent rainfall provides surface moisture for breeding sites formation, replenishes existing ponds, and promotes egg hatching success and immature survivorship [105,106]. Furthermore, vector populations and malaria parasite transmission risk decreases with increasing altitude [59,121], due to cooler temperatures that do not support rapid *Anopheles* mosquito development and parasite replication. Transmission risk is also shown to drop in arid regions or during periods of drought compared to wetter regions and the rainy season [66,108,122].

#### Egg to adult survivorship due to rainfall (P_EA_R)

To model the probability of survival as a function of hydrologic conditions, we adopt a sigmoidal function, based on several studies on the survival of immature *Anopheles gambiae* as surface water level changes in the breeding site [105–107]. Chances of survival and successful completion of development increases with increasing surface water levels, here estimated by cumulative rainfall, but are near zero when hydrologic conditions are anomalously low and gradually plateaus when surface water levels are high. Survival due to water variability shows similar trends across the egg, larvae and pupae development stages, although with varying degrees of sensitivities. To limit model complexity, we model survivorship due to hydrologic changes during egg-adult development aggregately as:

$$P_{EA}(R)=\frac{1}{{1+e}^{-a.R(c.R_{D}-b.R)}}$$ (13)

where *a*.*R* relates the egg-adult sensitivity to surface water levels and *c*.*R*_*D*_ is the standardized anomaly of cumulative weekly rainfall data computed over *D* previous days for a catchment site. The standardized anomalies for a site are calculated relative to the baseline average weekly cumulative rainfall (over D days) received at that site during 2005–2019. The parameter *b*.*R* indicates the mean anomaly level at which 0.5 survival is expected. Prior ranges for the length of cumulative rainfall are informed by studies of rainfall activity and mosquito density [20,72,97,98]. The model form for moisture-regulated survival, and the prior ranges for the mean anomaly and the slope are derived from empirical fittings of anopheles egg survival and moisture changes (see supplement).

#### Egg to adult survivorship (P_EA_T) and development time (τ_EA_) due to temperature

Several studies on the immature stages of *Anopheles gambiae* have shown that overall survival and development rates of egg and pre-adult mosquitoes are linked with water habitat temperature through a unimodal relationship [7,8,27]. Survival and development rates are highest around 24–28°C and exposure to temperatures far from this optimal level results in death or delayed and disrupted development of mosquitoes into the next life stage. We allow the rate of immature survival (P_EA_) and duration of egg-adult stage (τ_EA_) to vary as follows:

$$P_{EA}(T_{w})=-0.00924{T_{w}}^{2}+0.453T_{w}+-4.77$$ (14)

$$\tau_{EA}(T_{w})={\left(0.000111T_{w}(T_{w}-14.7)\sqrt{34-T_{w}}\right)}^{-1}$$ (15)

where T_w_ is habitat water temperature. Water temperature of mosquito habitats highly correlates with surrounding ambient air temperature. However, air temperature can underestimate mosquito survival and development, as it can be up to 6°C cooler than breeding site water temperature. Thus, to estimate the water temperature of breeding sites we use a simple linear model as per [123,124], where T_w_ = k*T_air_ + ΔT. Here, *k* is the slope and ΔT is a constant >0 capturing all antecedent impacts that raise the energy balance of the breeding site. Estimates of *k*, which represents the strength of the relationship between T_air_ and T_w_ have been shown to vary from 0.5 to 0.9[123,124] with broad uncertainty, depending on location and size of the breeding sites. Rates of evaporation, wind conditions and season also affect the strength of the relationship between air temperature and water temperature of aquatic habitats [123–126]. As estimates of *k* exhibit large variability and high specificity to local features, we set *k to unity* and ΔT to a constant value of 2°C due to absence of information on the location, size and ambient features of aquatic habitats in the study region. However, sensitivity analyses were further conducted to examine the effect of various values for k and ΔT on mosquito density and malaria transmission (see supplemental text).

#### Mosquito biting rate (a)

Female mosquitoes require blood meals for egg development and will feed on blood hosts to obtain the proteins and calories needed to support development [127]. After eggs mature and are oviposited, surviving females can again seek a host for blood feeding and repeat this cycle of gonotrophy. The amount of time spent in blood meal digestion and egg development is strongly impacted by temperature [9,128] and does not appear to depend on infection status of mosquitoes in the egg generation state [129]. The temperature-dependent estimate of the mosquito biting rate is found by inversion of the duration of gonotrophy:

a(T)=0.017T−0.165 (16)

#### Sporogony (n)

*Plasmodium* parasites within infected mosquitoes must develop further into sporozoites to produce infections in humans [130–132]. The duration of sporogony (*n*, i.e. the extrinsic incubation period) is modulated by ambient temperature [9,133,134] in a nonlinear fashion similar to mosquito development (Eq 17). Under warm temperatures, mosquitoes spend less time incubating the parasite and are likely to have more infective bites, whereas under colder conditions mosquitoes may not live long enough to feed again and transmit the pathogen. The relationship depicting the influence of temperature on the development of the *Plasmodium* parasite within the vector can be summarized mathematically as [135,136]:

$$n(T)={\left(0.000112T(T-15.384)\sqrt{35-T}\right)}^{-1}$$ (17)

#### Population scaling parameter (s)

Environmental and population factors, such as proximity to mosquito breeding sites, area vegetation, human genetics and behavior, and mosquito biting behavior have been recognized for creating malaria transmission hotspots [61,137–139]. These transmission hotspots reflect the so-called 20/80 rule [140–143], common in infectious diseases, in which a small proportion (e.g. 20%) of the population bears most of the burden of transmission (80%) and contributes considerably more to the force of infection than surrounding communities in the same region [137,140,141]. Because of this heterogeneity, the effective number of individuals actively involved in malaria transmission may be lower than the total human population in a region. Therefore, to model the population size of the transmission system, we estimate an effective population size within the model as *P–*the product of scaling parameter *s* and *N*, the human census population.

#### Simulation of malaria incidence

The malaria vector exhibits strong and complex relationships with the meteorology and hydrology of its local environment, which influence its entomology and ability to transmit the malaria parasite. Laboratory and field experiments have elucidated and quantified several of these relationships mathematically. Combining the EIR, which allows for a simplified expression of mosquito entomology and malaria transmission, with data-assimilation and mathematical modeling approaches has the potential to improve simulation of malaria incidence and representation of the biophysical relationships underlying climate modulation of malaria transmission in implicit models.

In this study, we use the environmentally-driven EIR model framework to simulate local malaria transmission among populations in Rwanda. A parsimonious model of malaria transmission, driven by temperature and rainfall, is coupled with Bayesian inference. This combined model-inference system is then used to estimate local transmission parameters, malaria epidemiology, and to simulate malaria incidence, by tracking the number of newly infected individuals as the environmentally-driven transmission model evolves over time. Our analyses indicate that the climate-malaria model can accurately simulate malaria incidence across Rwanda’s diverse climate landscape, while estimating ento-epidemiological parameters supporting local malaria transmission.

#### Study region

The study region is Rwanda–a small, landlocked country in eastern sub-Saharan Africa. Rwanda has diverse terrain, with elevations up to 4200m above sea level in the north and west and as low as 900m above seas level in the east and south [144]. The large difference in geography is associated with distinct climate regimes across the country. The highlands are temperate and experience year-round temperatures that hover around 17.5°–19°C, which are slightly less suitable to mosquito and parasite activity [8,9,27], compared to the warmer tropical temperatures (20°–24°C) found across the lowlands. Two rainy seasons exist in the country, primarily because of the seasonal progression of the Inter-Tropical Convergence Zone (ITCZ)–a longer season lasting from February–June and peaking in April and a shorter one between September–December, peaking in November. Additionally, across the country rainfall varies distinctly, increasing from the east and southeast (~900mm annual average) to the west and northwest (~1500mm annual average) [52,144].

Malaria transmission in Rwanda occurs year-round, and has two seasons, one peaking around April–May and the other during November–December. These peaks in transmission correspond to the two peaks in the bimodal rainy season. Although the entire population is at risk of transmission, malaria varies geographically across the country. In the highlands in the west and north, the burden of malaria is lower compared to the lowlands in the south and east [36]. In Rwanda, as is in most of sub-Saharan Africa, *Plasmodium falciparum* is the prevalent cause of malaria disease [145–147]; and *Anopheles gambiae* sensu lato (*s*.*l*.), a highly efficient vector of the parasite [148,149] is the most common malaria vector across the country [55,150,151]. *Anopheles funestus* and other *Anopheles* species are also found throughout the region but in fewer numbers.

#### Malaria and climate data

Malaria data used in this study were acquired from the Rwanda, Ministry of Health (MoH) and are publicly available via the Rwanda Biomedical Center (RBC) web portal [152]. In an effort to improve control of malaria transmission and lower the burden of malaria, Rwanda established improved reporting and laboratory testing practices for suspected malaria and other infectious diseases beginning in 2011, as well as greater Insecticide-treated bed net (ITN) coverage, Indoor Residual Spraying (IRS), and community-level case management [36,52,56]. We obtained weekly reports of parasitologically-confirmed malaria incidence during 2016 to 2019 for 42 public health catchment areas. These datasets were used to train the climate-malaria model in conjunction with data assimilation approaches for each catchment site.

Weekly rainfall (2005–2019) and temperature (2006–2016) data were obtained from the publicly available [153] University of California, Santa Barbara Climate Hazards Group InfraRed Precipitation and Temperature with Station dataset (CHIRPS and CHIRTS) products [154,155]. These data were then re-gridded to match each catchment site. Due to a lack of up-to-date data, weekly temperature averages derived from the 2006–2016 CHIRTS data were used to drive the climate-malaria model in conjunction with contemporaneous weekly CHIRPS rainfall estimates. Potential bias introduced from historical averages were assessed by comparing malaria incidence from final model conditions forced by available weekly temperature data versus historical averages (see supplement).

#### Model inference system

To simulate malaria transmission and infer model parameters, we pair the climate-malaria model with malaria incidence data using a data assimilation method for state-space models–the Ensemble Adjustment Kalman Filter (EAKF). Using Bayes’ Rule, the EAKF optimizes current model parameters and state variables, so that the first and second moments of the currently observed model state (i.e. incidence, μEI*E) align with those of the observed incidence data [156,157].

We initialize the model-EAKF system with an ensemble of 300 simulations with parameter values (D, μRS, μEI, a.R, b.R, q_R_ and s) and initial state estimates (S_0_, E_0_, I_0_, T_0_, R_0_) randomly drawn from uniform distributions, U (a, b), in which a and b indicate lower and upper boundary values (Table 2). We applied the EAKF using iterated filtering, which repeatedly assimilates the same time series [158] in a stepwise approach in order to improve convergence to more probable parameter and state variable solutions. Starting from time *t = 1*, the EAKF uses observation at t = 1 to update prior ensemble members into posteriors. To assimilate the next observation, the EAKF system is re-initialized from t = 1 using these same posteriors parameters as priors, filtering observations again from t = 1 to t = 2 and generating new posteriors. The model-EAKF system continues this recursive filtering and assimilation of observed data in a growing, stepwise fashion, until the final observation, t = 208, is assimilated, producing the filter estimates for the first iteration. A total of 10 iterations were performed using the observed time series, with subsequent iterations initialized with ensembles drawn from N (u, sigma) of the previous iteration. The annealing factor α_i_ used in computing sigma for each iteration *i*, is defined as follows:

$$\alpha_{i}=\left(1+\frac{0.5}{{\left(1+i\right)}^{0.75}}\right)$$ (18)

During data assimilation, divergence of the EAKF system from observed data can occur. To limit this, we inflate the variance (3%) and randomly re-probe (5%) the prior ensemble before each filter update [157]. Also, we observed that assimilation of periods of anomalously low malaria activity typically in the later years of the study resulted in massive failure of the EAKF during preliminary analysis. One explanation considered for these failures was the absence of data on intensified malaria control deployed across Rwanda, particularly in the last years of the study [36–38]. To ensure that the model-EAKF system is properly constrained by the data at hand, we evaluated the loglikelihood (*ll*_*t*_) of the full timeseries following each update to track filter failure (Eq 19). Filter failure was heuristically defined as a 5% departure from the current maximum loglikelihood. If this condition is violated, assimilation of an observation that results in massive failure is skipped and the prior is integrated forward to the next observation.


$$ll_{t}=-\frac{n}{2}\ln\;2\pi -\frac{n}{2}\ln\;\sigma^{2}-\frac{1}{2\sigma^{2}}\sum_{j=1}^{n}{(x}_{j}-{\mu )}^{2}$$ (19)

Where *x* is the reported malaria incidence at week *j*; *μ* is the ensemble mean and *σ* the ensemble standard deviation of malaria incidence simulations for week *j*, generated from posteriors following updates at time *t*.

**Table 2:** Description of the parameters of the malaria transmission model.

Parameter	Description	Prior ranges	Unit	Reference
*μ*_*BS*_ ^†^	Birth rate	(57*365)^-1^	day^-1^	Census
*δ* ^†^	Death rate	(53*365)^-1^	day^-1^	Census
*μ*_*EI*_ ^‡^	Duration of parasite incubation	7–14	day	Lit[45]
*μ*_*TS*_ ^†^	Duration of treatment + prophylaxis	30	day	Lit[78–83]
*μ*_*IR*_ ^†^	Duration of untreated infection	5	day	Lit[84–87]
f_T_^†^	Proportion of infected receiving full treatment	0.5	-	Lit[74]
*μ*_*RS*_ ^‡^	Duration of patent/sub-patent period	120–365	day	Lit[43,49,88–90]
P_MH_^†^	Probability of transmission from mosquito to human	0.5	-	Lit[50,91]
P_HM_^†^	Probability of transmission from human to mosquito	0.125	-	Lit[76,92,93]
q_R_^‡^	Infectivity of non-clinical cases relative to clinical cases	0–0.5 * (P_HM_)	-	Lit[46–48]
ε^†^	Number of eggs laid per gonotrophic cycle	50	-	Lit[94–96]
*μ*_*M*_ ^†^	Daily adult mortality rate	-ln 0.98	-	-
a.R^‡^	Egg-adult sensitivity to surface moisture	0–1	-	See supplement
b.R^‡^	Mean anomaly of accumulated rainfall	-5–5	-	See supplement
D^‡^	Length of accumulated rainfall contributing to mosquito breeding	7–200	day	Lit[20, 72, 97, 98]
s^‡^	Population scaling factor	0–1	-	-

^†^ Indicates model parameter values that are fixed based on literature or demographic data, whereas

^‡^ indicates parameter values that are inferred from model fitting of observed data.

For each local site, model fitting of malaria incidence is conducted inter-annually for four continuous years (2016–2019) and separately for each study year. Parameters for temperature-dependent vector and parasite biology were taken from literature, while the parameters for rainfall-dependent vector dynamics were estimated using data from field studies and fitting of the malaria transmission model. Inferred parameter values and uncertainty are reported as the mean and 95% credible interval (CI) of the posterior ensemble from the final iteration. Using these mean and 95% CI values, together with rainfall and temperature data, the transmission model was integrated forward to estimate the true state of observed malaria incidence and the uncertainty of that estimate. Model inference is not performed at the province level but aggregates of modeled incidence from local sites comprising the five provinces of Rwanda (East, Kigali City, South, West and North) are reported. We compared malaria incidence simulated by the model-EAKF system to observed local and regional data using Coefficient of Determination (R^2^) and Mean Absolute Relative Error (MARE). We also compare model inferred parameters to previous estimates from other studies.

To validate the model-inference system, we used synthetic malaria data generated with the model in free simulation. Parameter estimates inferred by the model-EAKF system using the synthetic data were then compared to the parameter values used to generate the synthetic data (see supplement). All simulations and analyses were conducted in R statistical software [159].

## Supporting information

S1 Text Synthetic inference testing and sensitivity analysis of the final model. **Fig A.** Model-EAKF simulated incidence of malaria from 2016–2019 for the 42 catchment sites. **Fig B.** Mosquito development and estimated survivorship as modulated by temperature and rainfall in model simulations. **Fig C.** Posterior parameters of the malaria transmission model estimated by the model-EAKF inference system during continuous simulation. **Fig D.** Comparison of the posterior parameters of the transmission model estimated by the model-EAKF system under continuous simulation and yearly initialization. **Fig E.** Average percent change in MARE for simulated malaria when individual entomological functions are decoupled from climate. **Fig F.** Relative error of mean posterior estimate of true parameters, over 10 iterations. **Fig G.** Percent change in RMSE of simulations of malaria incidence (left pane) and mosquito density (right pane) under various k and ΔT values relative to default model conditions. **Fig H.** Attack rate difference between simulated incidence generated across study sites using observed weekly temperature data and weekly average temperatures. **Fig I**. Difference of model predicted attack rate as proportion of full-treatment (f_T_) deviate from final model conditions. **Fig J**. Boxplots of average province-level attack rates of malaria incidence, predicted under continuous simulation. **Fig K**. The relationship between the malaria force of infection and model estimated weekly average EIR for sites found in the study provinces. **Table A.** Mean and 95% credible interval of posterior ensemble of model parameter estimates. **Table B.** Mean and 95% confidence interval estimates of *k*, the common rate of dispersion within the OEV for model-EAKF simulation.
(DOCX)
