# Supplementary material for: Inference and dynamic simulation of malaria using a simple climate-driven entomological model of malaria transmission
Source: PLoS Comput Biol. 2022 Jun 9;18(6):e1010161. doi: 10.1371/journal.pcbi.1010161 (PMC9182318; doi:10.1371/journal.pcbi.1010161)
Supplement: S1 Text — Fig A. Model-EAKF simulated incidence of malaria from 2016–2019 for the 42 catchment sites. Fig B. Mosquito development and estimated survivorship as modulated by temperature and rainfall in model simulations. Fig C. Posterior parameters of the malaria transmission model estimated by the model-EAKF inference system during continuous simulation. Fig D. Comparison of the posterior parameters of the transmission model estimated by the model-EAKF system under continuous simulation and yearly initialization. Fig E. Average percent change in MARE for simulated malaria when individual entomological functions are decoupled from climate. Fig F. Relative error of mean posterior estimate of true parameters, over 10 iterations. Fig G. Percent change in RMSE of simulations of malaria incidence (left pane) and mosquito density (right pane) under various k and ΔT values relative to default model conditions. Fig H. Attack rate difference between simulated incidence generated across study sites using observed weekly temperature data and weekly average temperatures. Fig I. Difference of model predicted attack rate as proportion of full-treatment (fT) deviate from final model conditions. Fig J. Boxplots of average province-level attack rates of malaria incidence, predicted under continuous simulation. Fig K. The relationship between the malaria force of infection and model estimated weekly average EIR for sites found in the study provinces. Table A. Mean and 95% credible interval of posterior ensemble of model parameter estimates. Table B. Mean and 95% confidence interval estimates of k, the common rate of dispersion within the OEV for model-EAKF simulation. (DOCX) [file pcbi.1010161.s001.docx]

**Supplemental figures and tables**

**Fig A in S1 Text**. Model-EAKF simulated incidence of malaria from 2016-2019 for the 42 catchment sites. In red dots and lines, with red shading are per capita incidence simulations of malaria transmission and the 95% credible interval (CI) estimated by the model-EAKF system. Reported incidence are shown as gray dots.


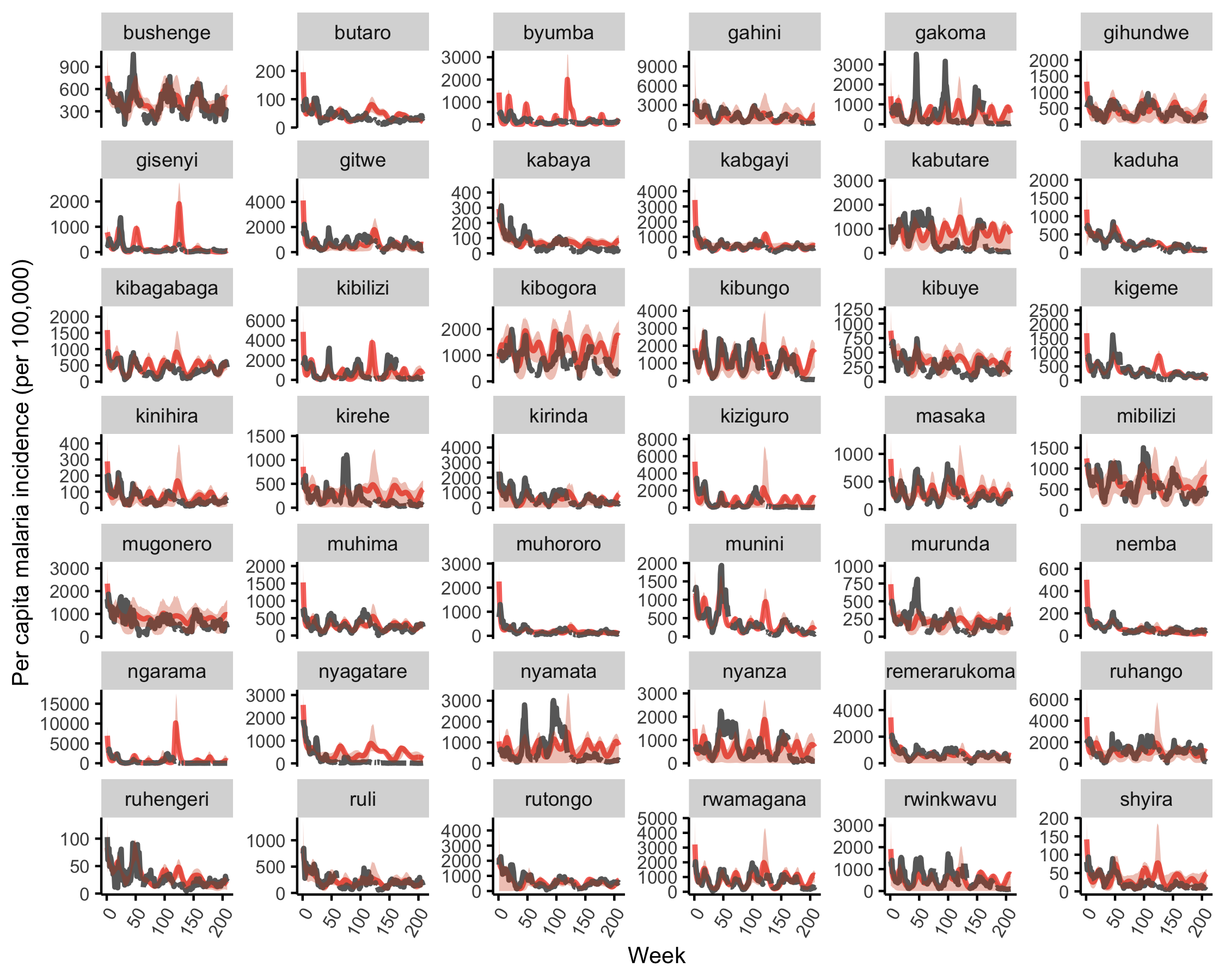


**Fig B in S1 Text**. Mosquito development and estimated survivorship as modulated by temperature and rainfall in model simulations. Colored lines and points indicate the aggregated estimate of duration of gonotrophy, duration of subadult development, extrinsic parasite incubation, temperature-regulated egg-adult survival, moisture-dependent egg–adult survivorship, mosquito density, entomological inoculation rate, for sites within the East(blue), Kigali City(yellow), North(grey), South (red) and West provinces (light blue).


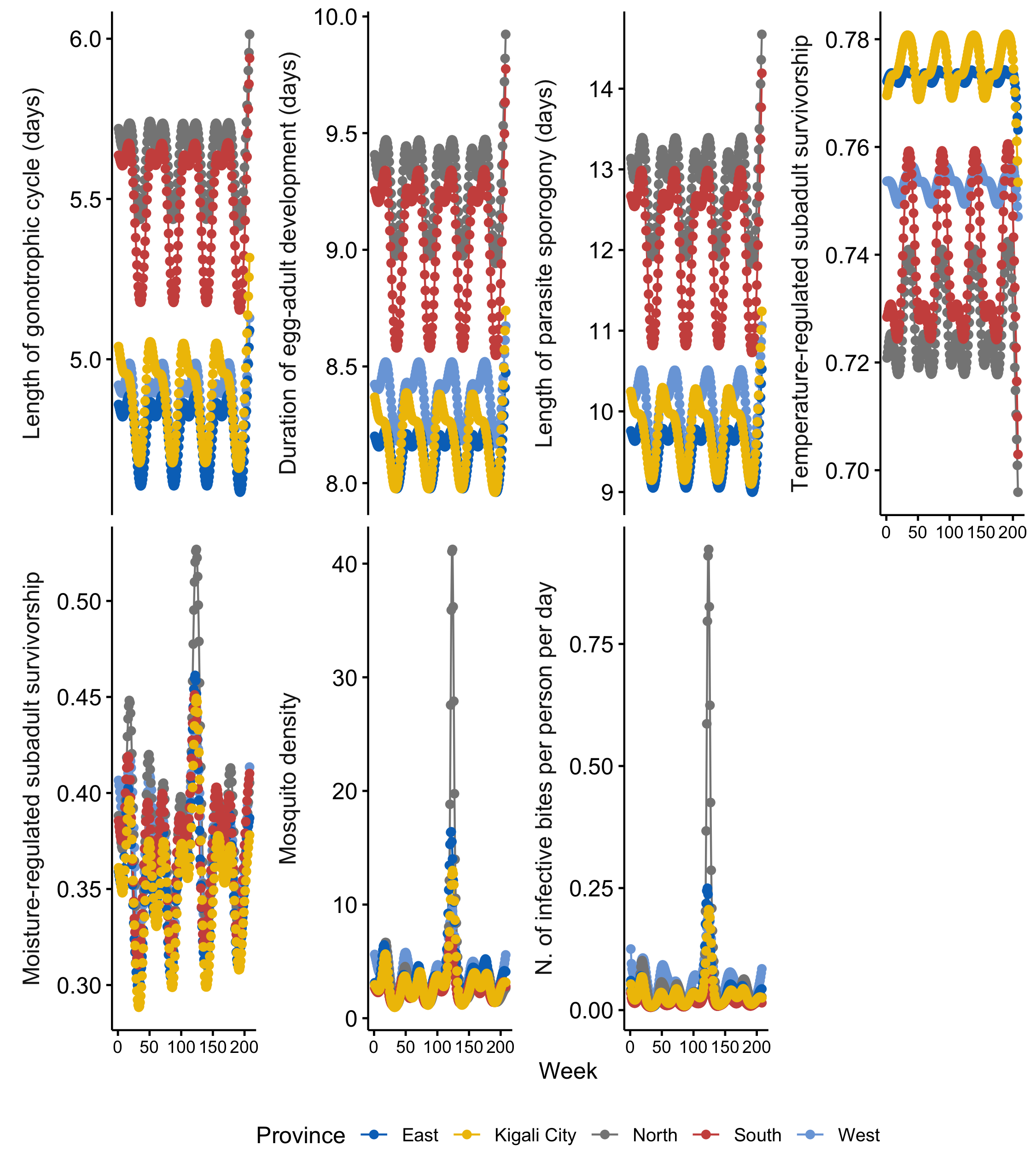


**Fig C in S1 Text**. Posterior parameters of the malaria transmission model estimated by the model-EAKF inference system during continuous simulation. Shaded density plots show spread of the estimated parameter values for sites in all of Rwanda(blue) and for those in East(yellow), Kigali City(gray), North(rose), South (light blue) and West (grayish blue) provinces, respectively. Jittered dots indicate the actual values, and the solid vertical lines mark the 12.5^th^ and 87.5^th^ percentiles, representing a majority (75%) of the sites.


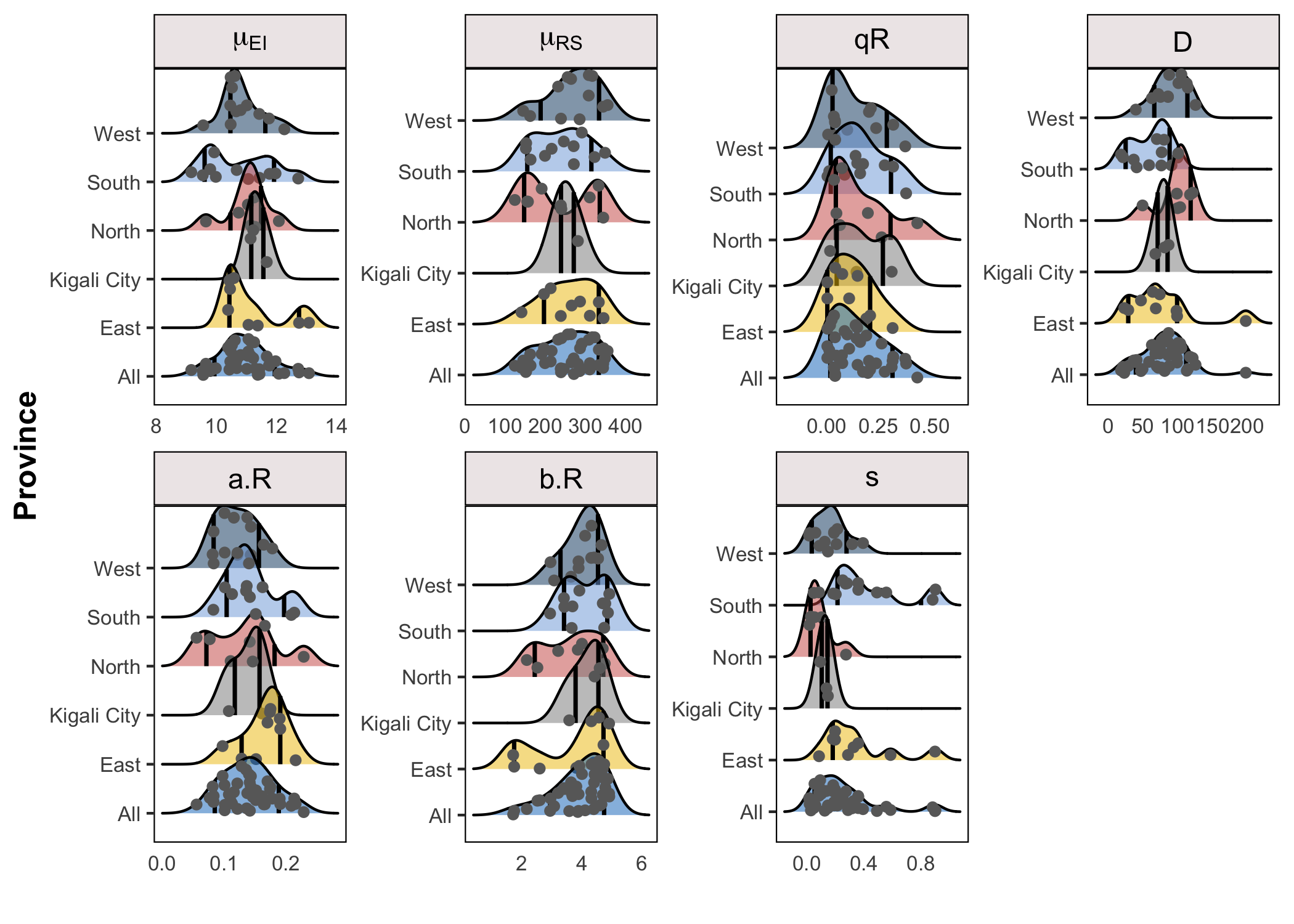


**Fig D in S1 Text**. Comparison of the posterior parameters of the transmission model estimated by the model-EAKF system under continuous simulation and yearly initialization. Boxplots are showing the median (thick line), interquartile range (box bounds), Q1 -1.5*IQR (lower whiskers), Q1 +1.5*IQR (upper whiskers) of the mean parameter values (row panels) inferred for each study site located across the five study provinces in Rwanda (column panels) when model inference is conducted continuously from 2016-2019 (purple) and when inference is initialized separately each year (yellow).


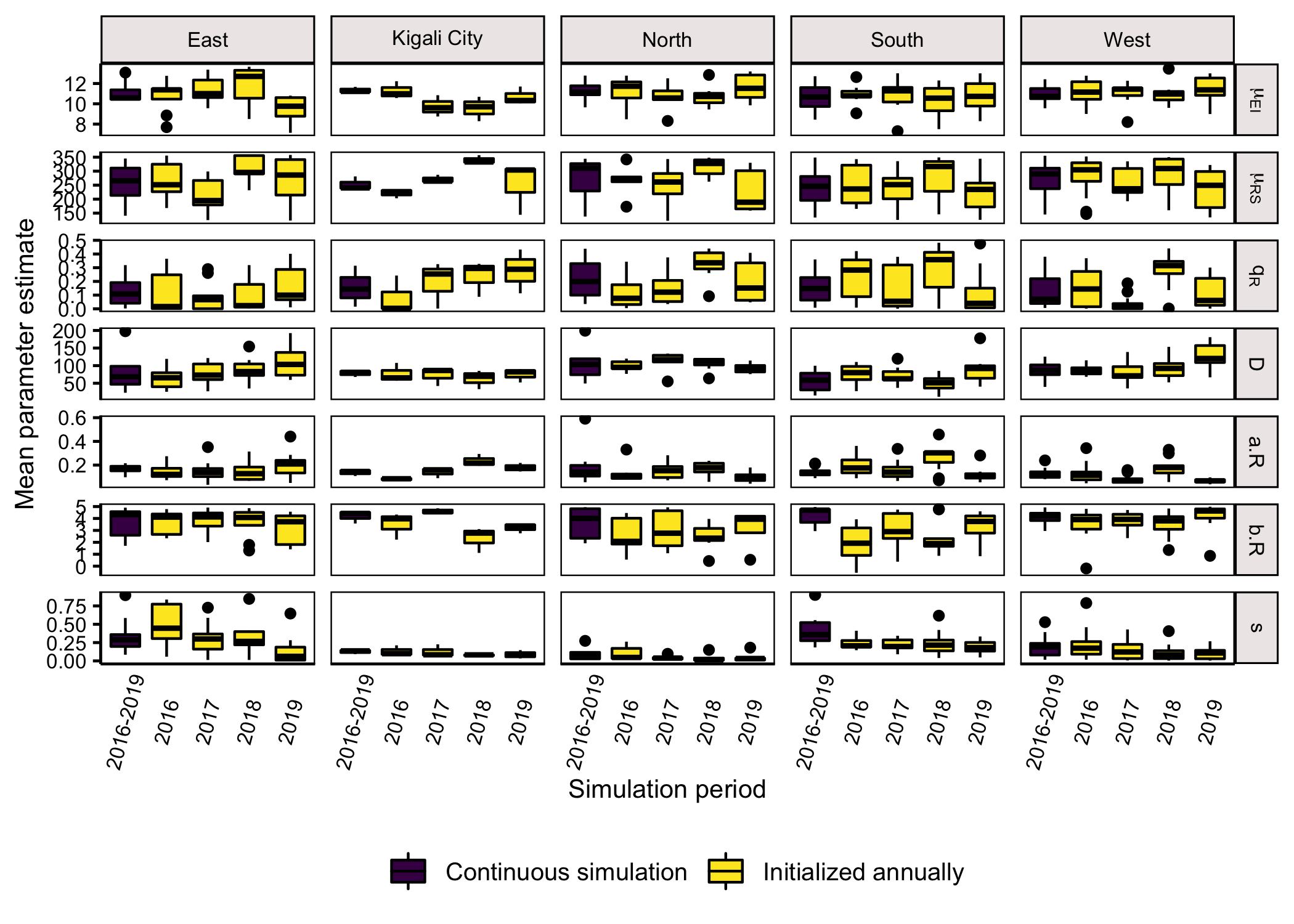


**Table A in S1 Text**. Mean and 95% credible interval for posterior ensemble of model parameter estimates.

| **Province** | **Site** | **Mean and 95% Credible Interval of Posterior Model Parameters** | | | | | | |
| --- | --- | --- | --- | --- | --- | --- | --- | --- |
|  |  | **µ_EI_** | **µ_RS_** | **q_R_** | **D** | **a.R** | **b.R** | **s** |
| **East** | Gahini | 10.58 (7.11–13.98) | 333.28 (189.28–365) | 0.21 (0.12–0.46) | 98.19 (79.71-114.46) | 0.19 (0.15–0.24) | 3.64 (2.56–4.77) | 0.9 (0.59–1) |
|  | Kibungo | 10.45 (7–13.73) | 310.9 (214.45–365) | 0.32 (0.04–0.44) | 47.06 (29.5-66.51) | 0.17 (0.15–0.21) | 4.89 (4.54–5) | 0.33 (0.17–0.55) |
|  | Kirehe | 10.45 (7.31–13.66) | 238.61 (135.82–350.37) | 0.11 (0.02–0.24) | 99.02 (20.63-186.57) | 0.15 (0.04–0.26) | 4.13 (2.47–4.96) | 0.08 (0.04–0.14) |
|  | Kiziguro | 10.43 (7.47–14) | 345.08 (251.06–365) | 0.04 (0–0.14) | 63.95 (50.03-77.88) | 0.22 (0.17–0.29) | 2.54 (1.91–3.11) | 0.36 (0.24–0.48) |
|  | Ngarama | 13.08 (10.06–14) | 140.74 (120–171.11) | 0 (0–0.01) | 23 (12.13-39.19) | 0.19 (0.13–0.27) | 1.73 (1.32–2.04) | 0.59 (0.39–0.81) |
|  | Nyagatare | 12.75 (11.35–13.85) | 213.43 (171.37–252.29) | 0 (0–0) | 197.8 (195-199.95) | 0.1 (0.05–0.17) | 1.2 (–2.41–2.69) | 0.18 (0.15–0.21) |
|  | Nyamata | 10.39 (7.27–13.67) | 196.76 (121.12–311.57) | 0.08 (0–0.23) | 28.77 (10.77-52.77) | 0.13 (0.06–0.2) | 4.69 (3.9–5) | 0.2 (0.09–0.32) |
|  | Rwamagana | 11.07 (7.02–13.98) | 265.59 (137.51–365) | 0.15 (0.03–0.45) | 73.99 (54.59-96.18) | 0.17 (0.1–0.23) | 4.39 (2.43–5) | 0.29 (0.17–0.46) |
|  | Rwinkwavu | 11.37 (7.08–14) | 286.52 (174.4–355.05) | 0.19 (0–0.42) | 68.43 (48.14-95.38) | 0.17 (0.13–0.24) | 4.26 (2.81–4.86) | 0.2 (0.13–0.28) |
| **Kigali City** | Kibagabaga | 11.22 (7.28–14) | 238.85 (120–348.79) | 0.31 (0.09–0.5) | 81.03 (59.88-102.92) | 0.16 (0.12–0.22) | 4.49 (3.24–5) | 0.15 (0.09–0.21) |
|  | Masaka | 11.14 (7.14–13.65) | 240.08 (130.74–365) | 0.02 (0–0.05) | 67.78 (46.95-93.47) | 0.11 (0.05–0.18) | 3.08 (–5–4.45) | 0.09 (0.05–0.14) |
|  | Muhima | 11.67 (7.91–14) | 281.2 (171.11–365) | 0.14 (0.07–0.29) | 86.77 (68.69-108.26) | 0.15 (0.1–0.2) | 4.29 (2.91–4.95) | 0.14 (0.07–0.22) |
| **North** | Butaro | 12.08 (9.06-14) | 124.22 (120-165.36) | 0.05 (0.03-0.08) | 99.63 (87.38-110.13) | 0.08 (0.07-0.09) | 4.75 (4.32-5) | 0.05 (0.04-0.07) |
|  | Byumba | 11.25 (7.67-14) | 190.95 (143.37-281.08) | 0.06 (0.02-0.13) | 98.67 (81.01-115.92) | 0.15 (0.1-0.19) | 2.74 (-5-4.1) | 0.05 (0.02-0.09) |
|  | Kinihira | 11.29 (7.94–13.83) | 310.59 (238.08–363.5) | 0.07 (0.01–0.14) | 103.48 (81.26-126.18) | 0.14 (0.07–0.22) | 3.9 (2.76–4.62) | 0.03 (0.02–0.04) |
|  | Nemba | 10.74 (7.2–14) | 156.84 (120–218.91) | 0.04 (0.02–0.06) | 49.32 (37.47-62.61) | 0.06 (0.04–0.07) | 4.65 (4.01–5) | 0.1 (0.07–0.15) |
|  | Ruhengeri | 10.81 (7.4-13.96) | 231.21 (131.05-342.17) | 0.37 (0.22-0.5) | 106.2 (92.3-121.02) | 0.13 (0.08-0.2) | 3.35 (1.91-4.42) | 0.03 (0.02-0.04) |
|  | Ruli | 11.05 (7.76–13.92) | 344.36 (312.22–364.23) | 0.2 (0.04–0.41) | 117.55 (93.51-140.78) | 0.17 (0.07–0.25) | 1.88 (–0.05–3.01) | 0.08 (0.06–0.11) |
|  | Rutongo | 9.65 (7–13.05) | 332.75 (248.73–353.32) | 0.44 (0.27–0.5) | 121.38 (107.9-139.87) | 0.23 (0.18–0.29) | 2.46 (1.98–3.23) | 0.27 (0.2–0.39) |
| **South** | Gakoma | 9.98 (7.21–14) | 290.87 (165.09–365) | 0.18 (0–0.4) | 21.12 (12.57-39.23) | 0.21 (0.15–0.3) | 3.23 (1.1–4.64) | 0.18 (0.1–0.35) |
|  | Gitwe | 11.43 (7.83–14) | 270.66 (143.95–365) | 0.04 (0–0.1) | 76.85 (37.64-128.86) | 0.11 (0.07–0.17) | 4.65 (3.9–5) | 0.36 (0.22–0.54) |
|  | Kabgayi | 12.72 (8.75–14) | 348.75 (295.11–365) | 0.15 (0.09–0.26) | 69.83 (60.37-79.66) | 0.16 (0.15–0.18) | 3.04 (–5–4.56) | 0.25 (0.16–0.36) |
|  | Kabutare | 11.97 (8.28-14) | 163.33 (135.19-213.38) | 0.38 (0.25-0.5) | 76.3 (56.22-95.31) | 0.14 (0.1-0.18) | 3.38 (2.95-3.85) | 0.2 (0.04-0.43) |
|  | Kaduha | 9.55 (7.17–13.91) | 151.62 (133.79–195.8) | 0.32 (0.21–0.43) | 90.96 (82.42-98.97) | 0.12 (0.11–0.13) | 4.91 (4.61–5) | 0.49 (0.34–0.76) |
|  | Kibilizi | 9.93 (7.01–13.19) | 246.13 (132.38–365) | 0.28 (0.09–0.5) | 18.38 (10-31.42) | 0.21 (0.14–0.27) | 4.77 (4.38–5) | 0.56 (0.27–0.91) |
|  | Kigeme | 11.77 (7.86–13.58) | 199.23 (168.93–242.22) | 0.01 (0.01–0.02) | 58.69 (47.16-71.39) | 0.1 (0.09–0.11) | 3.68 (3.48–3.98) | 0.9 (0.65–0.95) |
|  | Munini | 9.79 (7-12.5) | 152.44 (120-207.54) | 0 (0-0.01) | 36.69 (26.81-47.37) | 0.08 (0.08-0.09) | 4.86 (4.55-4.98) | 0.88 (0.65-1) |
|  | Nyanza | 10.67 (7.39–13.69) | 215.82 (123.05–336.33) | 0.16 (0.01–0.37) | 40.82 (13.63-72.75) | 0.14 (0.08–0.21) | 4.72 (4.23–5) | 0.27 (0.14–0.4) |
|  | Remera rukoma | 9.16 (7–13.39) | 322.72 (219.07–365) | 0.14 (0–0.34) | 99.69 (87.98-113.14) | 0.14 (0.11–0.17) | 3.38 (3.06–3.77) | 0.28 (0.2–0.4) |
|  | Ruhango | 11.06 (7.72–13.87) | 271.14 (156.79–365) | 0.09 (0.01–0.2) | 80.16 (26.77-139.67) | 0.14 (0.03–0.28) | 2.76 (1.26–3.7) | 0.36 (0.21–0.52) |
| **West** | Bushenge | 10.52 (7.17–14) | 285.44 (184.48–365) | 0.03 (0.02–0.06) | 85.94 (50.77-124.04) | 0.08 (0.05–0.13) | 2.55 (–5–4.05) | 0.15 (0.09–0.22) |
|  | Gihundwe | 10.81 (7.25–13.88) | 317.09 (232.56–363.03) | 0.04 (0.01–0.07) | 39.61 (22.01-58.32) | 0.12 (0.04–0.19) | 3.82 (2.92–4.4) | 0.32 (0.24–0.41) |
|  | Gisenyi | 10.47 (7.14-13.75) | 255.66 (133.47-353.19) | 0.02 (0.01-0.05) | 114.38 (74.38-154.09) | 0.16 (0.1-0.23) | 4.62 (4.04-4.99) | 0.12 (0.06-0.21) |
|  | Kabaya | 9.56 (7–12.82) | 355.16 (300.52–365) | 0.38 (0.17–0.5) | 125.56 (106.03-161.23) | 0.14 (0.11–0.18) | 3.71 (–2.63–4.73) | 0.02 (0.01–0.03) |
|  | Kibogora | 11.43 (8.08–14) | 162.31 (120–256.69) | 0.2 (0–0.45) | 75.13 (26.37-133.46) | 0.14 (0.08–0.21) | 4.44 (3.34–5) | 0.21 (0.14–0.28) |
|  | Kibuye | 10.47 (7.06–13.9) | 264.26 (170.81–351.87) | 0.21 (0.02–0.46) | 100.94 (25.63-187.92) | 0.14 (0.07–0.22) | 4.25 (2.72–4.99) | 0.09 (0.06–0.12) |
|  | Kirinda | 11.75 (7.78–14) | 343.91 (244.88–365) | 0.32 (0.15–0.5) | 85.83 (67.66-102.87) | 0.18 (0.16–0.21) | 4.51 (3.98–4.78) | 0.39 (0.3–0.55) |
|  | Mibilizi | 10.46 (7.52–13.54) | 308.06 (231.66–363.15) | 0.04 (0.01–0.07) | 62.75 (25.81-100.91) | 0.1 (0.03–0.18) | 2.74 (1.06–3.78) | 0.19 (0.11–0.28) |
|  | Mugonero | 10.61 (7.12–13.9) | 232.01 (130.67–352.91) | 0.24 (0.03–0.47) | 105.17 (23.24-192.28) | 0.12 (0.02–0.22) | 4.06 (1.6–4.96) | 0.19 (0.13–0.26) |
|  | Muhororo | 12.26 (8.03–13.78) | 145.01 (120.64–199.82) | 0.1 (0.06–0.16) | 72.02 (59.51-85.13) | 0.1 (0.09–0.12) | 3.59 (3.21–4.22) | 0.21 (0.14–0.3) |
|  | Murunda | 10.66 (7.05–13.97) | 239.05 (156.38–347.02) | 0.04 (0–0.09) | 112.91 (13.85-198.92) | 0.08 (0.02–0.15) | 4.04 (1.53–4.96) | 0.06 (0.04–0.08) |
|  | Shyira | 11.01 (7.38–13.87) | 308.39 (230.14–364.51) | 0.01 (0–0.02) | 87.63 (49.86-124.96) | 0.08 (0.01–0.15) | 3.59 (1.26–4.72) | 0.02 (0.01–0.02) |

**Fig E in S1 Text**. Average percent change in MARE for simulated malaria when individual entomological functions are decoupled from climate. A function is decoupled from temperature or rainfall by setting the function to a constant value, determined by annual temperature or rainfall for each year. Barplots show the average percent change in model fitness across all sites, as a resulting of seasonal decoupling of temperature-regulated sporogony, surface moisture-dependent egg–adult survival (P_EAR_), temperature-regulated mosquito biting rate (a), temperature-regulated lifetime number of eggs (B), temperature-dependent egg–adult survival (P_EAT_) and temperature-regulated duration of subadult development time(τ_EA_).


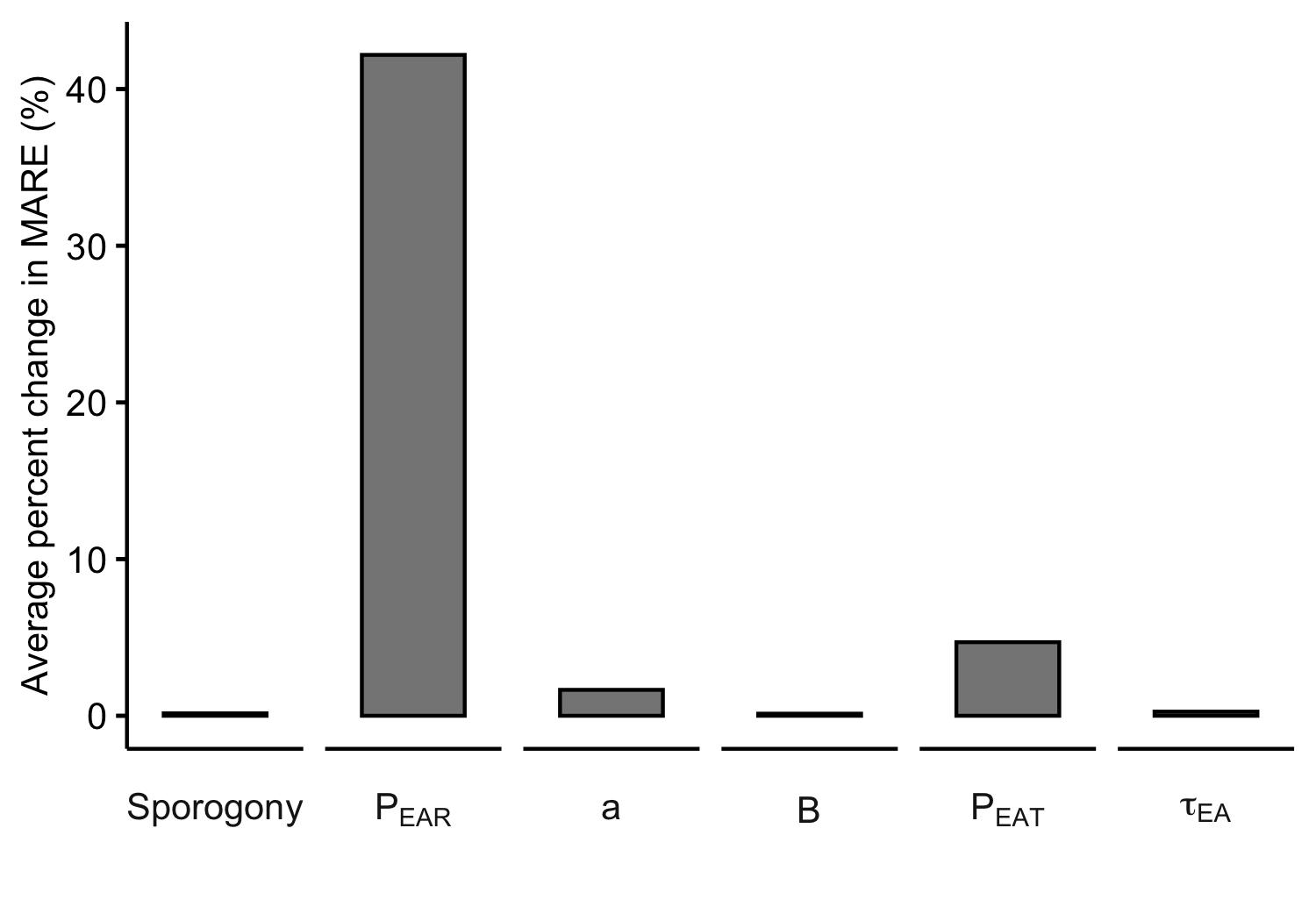


**Supplemental text**

**Synthetic testing**

Here, we assess the identifiability of the model-EAKF inference system using ‘synthetic’ observations generated with the model in free simulation.

*Synthetic incidence*

Fifteen sets of prescribed model parameters and initial state conditions were used as truths in this experiment. These sets of parameters were identified from a randomly generated set (N=1000) as those that produced free simulations of malaria incidence similar to seasonal malaria in Kibungo and with attack rates within ranges typically observed in the Rwanda malaria data (2016-2019, across all sites). Using rainfall and temperature data from 2016 for the Kibungo catchment site, along with the 15 sets of parameters, the climate-driven model was integrated starting from week 1, at a weekly time step, for 52 weeks under free simulation. Weekly incidence of malaria (µ_EI_*E) during these simulations was recorded.

*Data assimilation and inference*

For data assimilation using the EAKF, observational error variance (OEV) of the data must be defined. For the synthetic experiments, OEV is defined assuming the malaria data are overdispersed and follow a negative-binomial specification:

$$OEV_{t}=\gamma_{t}+\frac{\gamma_{t}^{2}}{\gamma_{t}^{k}}$$

where $\gamma$ is the true incidence at time $t$, and $k$ is the common rate of dispersion as $\gamma$ changes over time. For the synthetic analysis, $k$ was set to a constant value of 0.5. To generate observed incidence for each true incidence, five realizations are taken from NB ($\gamma_{t}$, $\frac{1}{\gamma_{t}^{k}}$) and paired separately with the model-EAKF system for data-assimilation.

With an ensemble of 300 simulations initialized with randomly drawn parameters and initial state conditions, we applied the model-EAKF inference system in an iterated approach (see main text). 10 iterations over each full timeseries of synthetic malaria incidence data were conducted. The ensemble mean of the final posterior, *x*, was the estimate for each model parameter. For each of the five stochastic realizations of the ‘true’ synthetic malaria incidence, the model-EAKF was re-initialized five times, each time with a new ensemble of 300 parameters and state variables, resulting in total of twenty-five estimates per parameter. The relative error (RE) of final parameter estimates *x* of each true parameter, inferred from realization *j*, during re-initialization *i*, is calculated as follows:

$$RE_{ij}=\frac{truth-x_{ij}}{truth}$$

Results from synthetic testing, shown in Fig. S6, indicate the EAKF inference system is capable of capturing the underlying parameters of transmission for the climate-forced malaria transmission model. For all 15 sets of true parameters, posterior error was substantively reduced, although variation in posterior accuracy was observed for some parameters.

**Fig F in S1 Text**. Relative error of mean posterior estimate of true parameters, over 10 iterations. Each histogram represents the spread of RE for each parameter (column panels) from 5 realizations and 5 re-initializations for each synthetic truth (row panels). The median RE is shown in solid black lines, and dashed black lines indicate the 1^st^ (25^th^ percentile) and 3^rd^ quartiles (75^th^ percentile) of the RE. Solid red line indicates zero error relative to the truth.


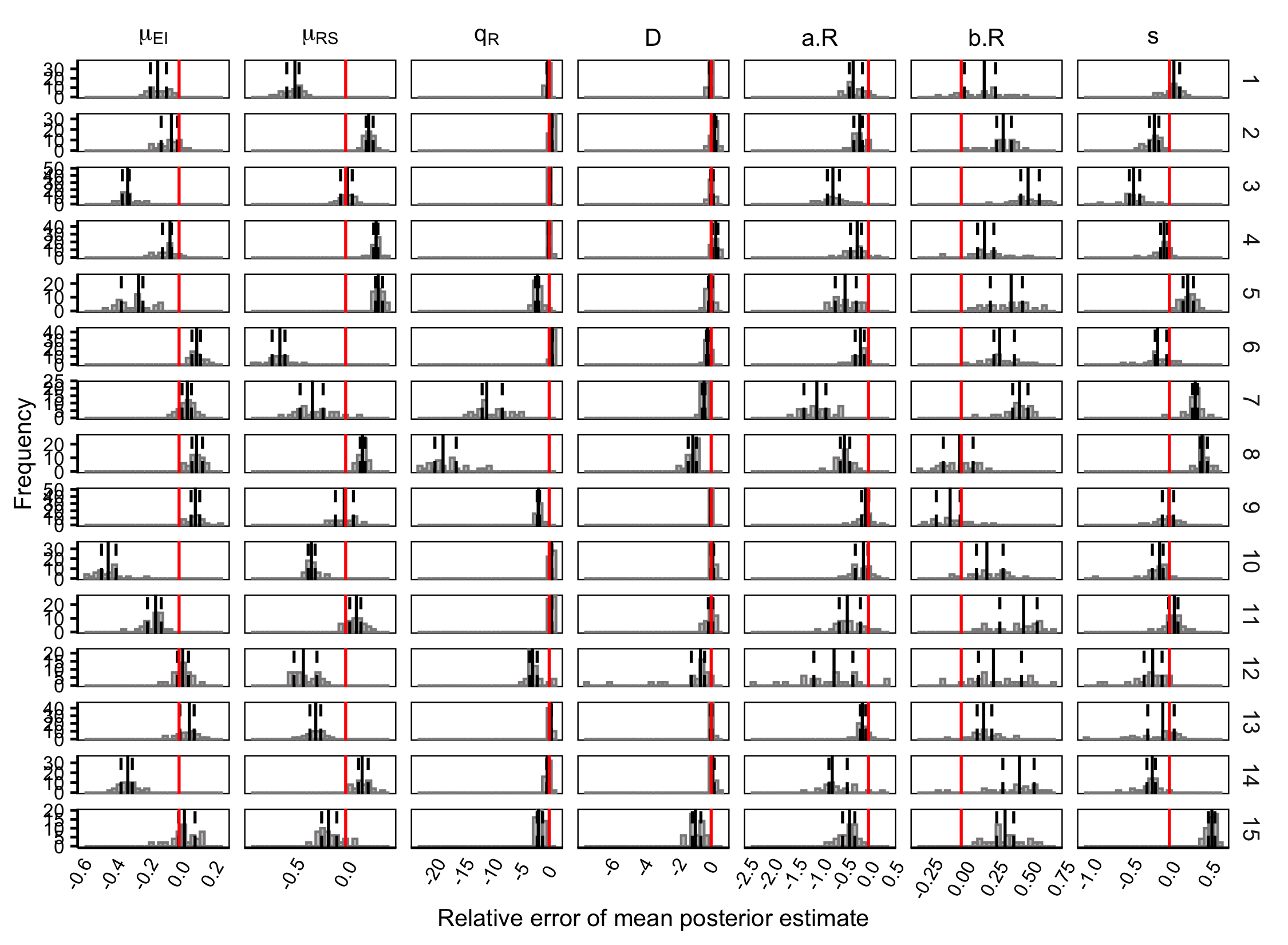


**Observation Error Variance (OEV) of malaria data**

For observations of malaria incidence across study sites, we assumed the data are over-dispersed and follow a negative binomial distribution. We define the OEV of weekly malaria for the model-EAKF system as follows:

$$OEV_{t}=\gamma_{t}+\frac{\gamma_{t}^{2}}{\gamma_{t}^{k}}$$

Given that true incidence is unknown, $\gamma_{t}$is taken as the response at time $t$ from a cubic spline smoothing of the full malaria timeseries for a site; and $k$ is the common aggregation parameter for observing malaria incidence. Maximum likelihood estimates of $k$ (Table S2) were determined for each site by numerical maximization of the log-likelihood function[1], using batch-gradient optimization within the optim function in R[2].

The OEV represents the variance of the true mean of observed cases, as caused by random measurement error of the unknown mean. Issues such as poor surveillance catchment and high rates of under-reporting that could lead to significant shifts of the measured mean are not explicitly accounted for by the OEV, but instead handled as systemic bias. Surveillance of malaria and other serious infectious diseases has significantly improved in Rwanda. This owes to major government undertakings [3,4] to transform the health system, eliminate malaria, and improve healthcare accessibility, especially through universal health insurance that enables individuals to visit and receive treatment for malaria without cost barriers[5]. In addition to the regional health center, local public health facilities and health posts that make up the passive surveillance network, active surveillance is set-up through Community Health Workers (CHW) at the Village administrative level throughout Rwanda [4]. These health personnel are specially trained to treat confirmed malaria cases at the household level and report them to the regional surveillance network. The comprehensive system in place in Rwanda, consequently, raised our confidence in the quality of observations and eliminated the need for explicit estimation of reporting rates or surveillance coverage. However, the effect of the surveillance network on modeled dynamics could be readily incorporated by $\rho*\mu_{EI}*E$. Here $\mu_{EI}*E$ is the true incidence of the transmission system and $\rho$ is the rate of under-reporting. Given the strong surveillance network in Rwanda $\rho$ was set to 1 within the transmission model.

**Table B in S1 Text**: Mean and 95% confidence interval estimates of $k$, the common rate of dispersion within the OEV for model-EAKF simulation.

| **Province** | **Site** | **Estimate (95% CI)** |
| --- | --- | --- |
| **East** | Gahini | 0.353 (0.285-0.421) |
|  | Kibungo | 0.317 (0.254-0.381) |
|  | Kirehe | 0.368 (0.297-0.439) |
|  | Kiziguro | 0.313 (0.244-0.383) |
|  | Ngarama | 0.365 (0.27-0.46) |
|  | Nyagatare | 0.473 (0.397-0.55) |
|  | Nyamata | 0.275 (0.211-0.338) |
|  | Rwamagana | 0.319 (0.254-0.385) |
|  | Rwinkwavu | 0.323 (0.253-0.393) |
| **Kigali City** | Kibagabaga | 0.395 (0.329-0.461) |
|  | Masaka | 0.374 (0.303-0.446) |
|  | Muhima | 0.444 (0.372-0.516) |
| **North** | Butaro | 0.758 (0.654-0.862) |
|  | Byumba | 0.435 (0.363-0.508) |
|  | Kinihira | 0.608 (0.509-0.707) |
|  | Nemba | 0.689 (0.595-0.783) |
|  | Ruhengeri | 0.739 (0.633-0.845) |
|  | Ruli | 0.598 (0.511-0.684) |
|  | Rutongo | 0.521 (0.447-0.594) |
| **South** | Gakoma | 0.271 (0.198-0.345) |
|  | Gitwe | 0.384 (0.315-0.453) |
|  | Kabgayi | 0.485 (0.419-0.552) |
|  | Kabutare | 0.45 (0.379-0.52) |
|  | Kaduha | 0.562 (0.481-0.642) |
|  | Kibilizi | 0.284 (0.215-0.354) |
|  | Kigeme | 0.493 (0.418-0.568) |
|  | Munini | 0.469 (0.396-0.541) |
|  | Nyanza | 0.382 (0.314-0.45) |
|  | Remera Rukoma | 0.449 (0.382-0.515) |
|  | Ruhango | 0.412 (0.345-0.478) |
| **West** | Bushenge | 0.609 (0.536-0.683) |
|  | Gihundwe | 0.566 (0.489-0.642) |
|  | Gisenyi | 0.372 (0.295-0.448) |
|  | Kabaya | 0.726 (0.618-0.834) |
|  | Kibogora | 0.432 (0.365-0.499) |
|  | Kibuye | 0.518 (0.442-0.594) |
|  | Kirinda | 0.443 (0.363-0.524) |
|  | Mibilizi | 0.441 (0.372-0.511) |
|  | Mugonero | 0.633 (0.55-0.716) |
|  | Muhororo | 0.651 (0.564-0.738) |
|  | Murunda | 0.514 (0.439-0.588) |
|  | Shyira | 0.775 (0.649-0.9) |

**Parameterization of moisture-regulated subadult survivorship**

Here, parameterization of mosquito-dependent subadult survivorship is described. The prior ranges indicating the period of cumulative rainfall (D) on subadult mosquito survivorship are based on reported understanding of rainfall activity and mosquito density[6–9]; in contrast, the prior ranges for the mean anomaly (b.R) and the slope (a.R) are informed by least-square fitting to *Anopheles* mosquito survival given changing surface moisture conditions [10].

Under laboratory conditions Shililu et al. exposed equal numbers of eggs of field-caught *Anopheles* *gambiae* mosquitoes to soil with different soil moisture levels[10]. After a pre-defined number of days (1, 5, 10, 15), the soil moisture content and proportion of eggs hatching to larvae under the different settings were assessed. The resulting relationship between soil moisture level and egg survival to larvae stage was fitted to:

$$H=\frac{1}{{1+e}^{-a.R_{0}(m_{std}-b.R_{0})}} (18)$$

where *H* is the age-adjusted larval hatching success of eggs under moisture level *m_std_,* which is computed as the standardized anomaly of experimental soil moisture conditions, *m*, a.R is the sensitivity of eggs to moisture, and b.R is the mean moisture level at which 50% survivorship is observed. The value of *m* used in Eq. 18 is derived from the logistic function below (Eq 19) that relates the soil moisture content remaining given the length of the experiment in days[10]:

$$m=m_{0}e^{-d} (19)$$

Here *m_0_* is set to the known moisture content (20%) at the start of the experiment (d=0) and λ is the moisture loss rate during the experiment and is estimated by least-squares as 0.1028 (99% CI= 0.0903 – 0.1153), with *R* (Pearson Correlation) = 0.9888.

Finally, adjusting for the effects of aging on egg survival [10] given the length of days under desiccation, we defined the survivorship due to soil moisture levels as $H=H_{0}^{1/d}$, where *H_0_* is the observed proportion of anopheles eggs hatching following *d* days of desiccation. Using least-square fitting of Eq. 18 relating *H* and *m*, *a.R*_0_ = 0.3296 (99% CI= 0.0137 – 0.6455), and b.R_0_ = -2.980 (99% CI= -5.7167 – -0.2425), with R (Pearson Correlation) = 0.7514. Based on preliminary analysis from malaria simulations for select sites showing poor fitting, we slightly relaxed the bounds of uncertainty on *a.R*_0_ and b.R_0_ to 0 – 1, and -5 – 5, respectively, to account for the limited range of anomalous moisture conditions to which experimental mosquitoes were exposed.

**Posterior estimates of model parameters**

The model-EAKF system also inferred a number of system parameters not discussed in the main text. Estimates of the length of accumulated rainfall conditions contributing to mosquito habitat levels (D) were generally in agreement throughout Rwanda. For sites in the East, Kigali City, North, South, and West provinces (Fig. S3 and Table S1), the median duration of the influence for cumulative antecedent rainfall conditions was 68, 81, 101, 70, and 87 days, respectively. Though previous studies relating cumulative rainfall conditions to mosquito habitat level are unknown in the region, these estimates are within ranges of previous estimates of antecedent rainfall conditions strongly correlating with mosquito density and malaria activity[6–9]. The initial prior ranges of parameters governing sub-adult survivorship due to moisture conditions (a.R and b.R) were taken from fittings of experimental data[10] on sub-adult survivorship (see supplement). Based on these estimates, the sub-adult mosquito sensitivity to surface moisture conditions, a.R_,_ ranged from 0.055– 0.23 and the mean anomaly of moisture, b.R, centered around 4.06 across Rwanda (Fig. S3).

Similarly, model estimates of the population scaling factor (s), which indicates the effective population size, were similar throughout Rwanda. For most sites (i.e., 75% of sites), the effective population size estimates were 0.15–0.35 of the census population (Fig S3). However, some sites, specifically, in the South and East regions, had effective population sizes of 0.5 or more of the census population. In contrast, sites located in the North and West provinces typically had a lower scaling factor (Table S1). These estimates suggest more focal transmission in regions that are less climatically suitable and indicate generally a small proportion of the total population involved in malaria transmission.

**Water temperature (Tw) sensitivity of the malaria model**

To estimate the water temperature of breeding sites, we use a simple linear model, as per [11,12], with T_w_ = *k**T_air_ + ∆T, where *k* is the slope and ∆T is a constant >0 representing antecedent effects. Breeding site temperatures can be up to 6°C warmer than surrounding ambient air. Additionally, the magnitude of the association between T_air_ and T_w_ has been shown to vary from 0.5 to 0.9 [11,12], depending on water pool size and ambient location features. In the main text, we set *k=1* and ∆T to a constant value of 2°C. Here, we evaluate the sensitivity of the malaria model to changing the water temperature model. A total of 500 random combinations of k and ∆T were drawn using Latin Hypercube Sampling (LHS) with the boundary values [0,1] and [1, 6], respectively. The malaria model for each catchment site was integrated to the end of the study period using the randomized Tw conditions and the mean posterior parameter estimates from the model-EAKF system. We subsequently computed the relative change in mosquito density and RMSE of simulations of malaria incidence with the randomized Tw conditions compared to the main model.

Analyses indicate that malaria incidence simulations generated with the optimized model were generally insensitive to changes of k and ∆T. Model fit to malaria incidence across the 42 local catchment sites on average did not deteriorate markedly and changes to mosquito density were moderate as values of k and ΔT varied (Fig S7). Improvements to model fit outside the default conditions were slight on average– about 5%, occurring typically at increasingly larger values of ΔT and increasingly lower values of k.

**Fig G in S1 Text**. Percent change in RMSE of simulations of malaria incidence (left pane) and mosquito density (right pane) under various k and ΔT values relative to default model conditions. Zero indicates no change in RMSE as k, the slope, and ΔT deviate from the default parameters of the Tw model; Tw = k*T_air_ + ΔT, where k=1 and ΔT=2.


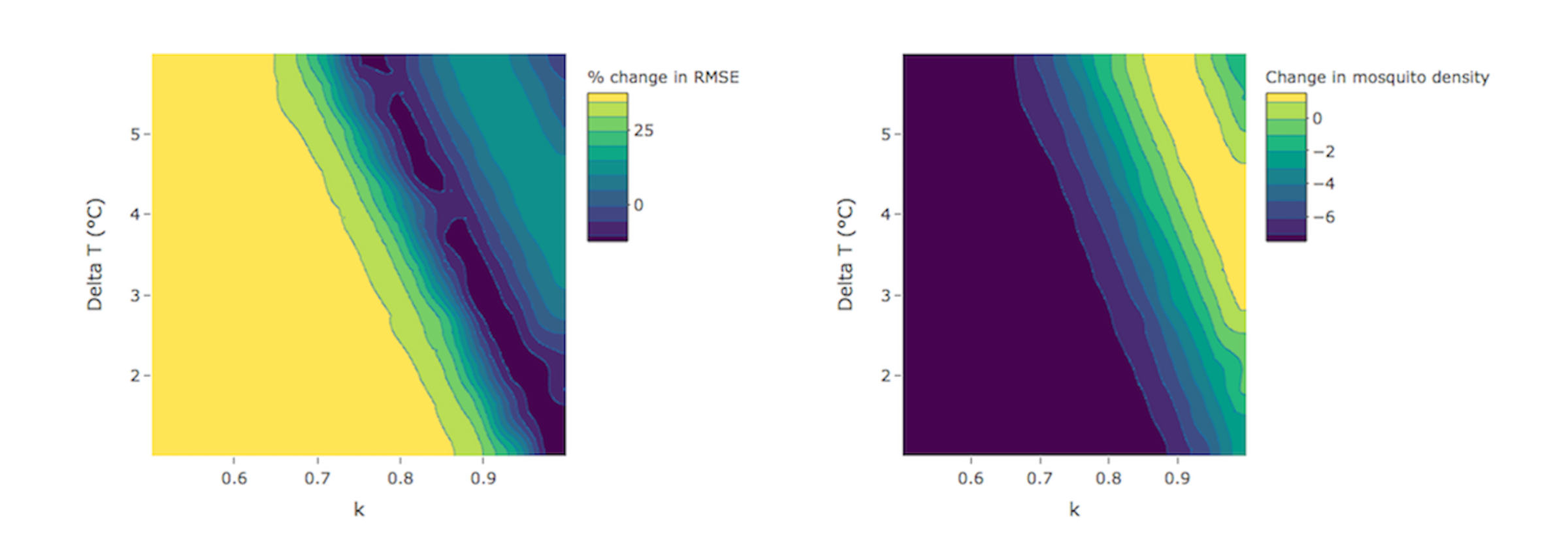


**Model sensitivity to historical temperature averages.**

Here, we assess potential simulation and estimation bias resulting from the use of weekly historical average temperatures rather than contemporaneous weekly observations of temperature. During preliminary testing, we observed that only the CHIRTS data, among several openly-available remotely-sensed temperature datasets, produced transmission outcomes similar to levels observed in the study region. In the final model, temperature climatology based on 2006–2016 CHIRTS data was used to simulate transmission for the study period (2016–2019), as concurrent temperature data for the CHIRTS temperature dataset were not available. The analysis below evaluated the potential influence of weekly temperature variability on annual malaria attack rate for the 42 regions of the study.

For each site, using the final optimized model parameters, simulations were generated for a set of 5 randomly selected consecutive four-year period between 2006 and 2016, for which weekly CHIRTS temperature and CHIRPS rainfall data were available[13,14]. Alternate simulations for each of the five four-year periods were subsequently generated using weekly average temperature based on 2006-2016. The difference in the annual attack rate between the simulations generated using temperature observations and averages are reported below. Results indicate that use of weekly average temperature conditions does not significantly bias simulations of malaria incidence (Fig. S8). Generally, sensitivity to historical temperature averages was low, though a few exceptions were observed.

**Fig H in S1 Text**. Attack rate difference between simulated incidence generated across study sites using observed weekly temperature data and weekly average temperatures.


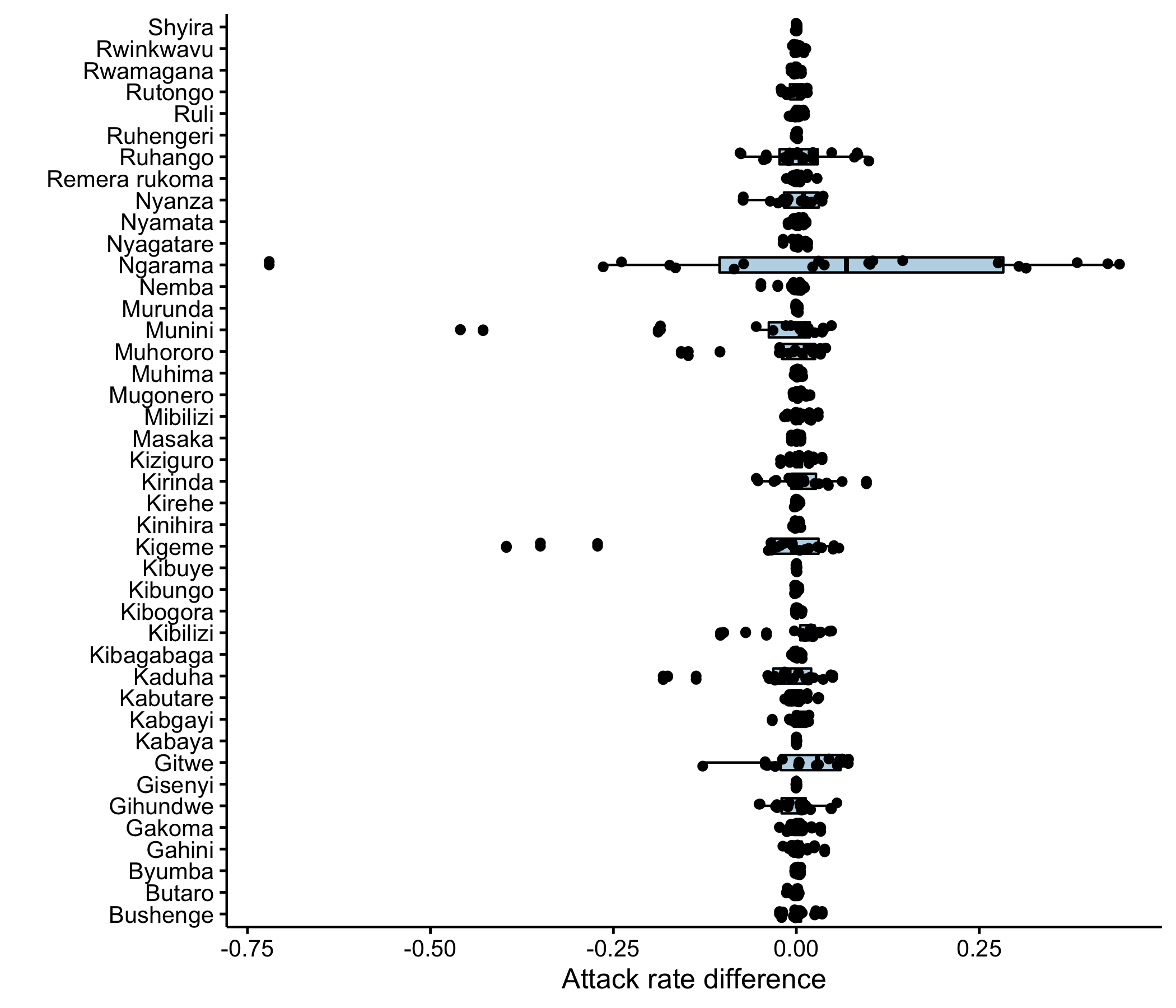


**Sensitivity of the final model to full treatment rate (f_T_)**

Based on the recent Rwanda DHS survey, which found that 42.24 – 63.98 % of individuals with fever seek treatment, we fixed the proportion of malaria cases receiving full treatment (f_T_) within the model to 0.5, i.e., 50%. In the analysis below, we varied the value of f_T_ between 0.25 – 0.75 in the final model and evaluated the change to predicted malaria attack rate as f_T_ changes, relative to the final model. Analyses suggest that model predicted malaria incidence shows a slight sensitivity to changing rates of full treatment across the various study locations (Fig. S8). However, even at the extreme low and high rates of treatment tested here, the difference in malaria attack rate remained fairly low, though a few sites saw larger deviations.

**Fig I in S1 Text**. Difference of model predicted attack rate as proportion of full-treatment (f_T_) deviate from final model conditions.


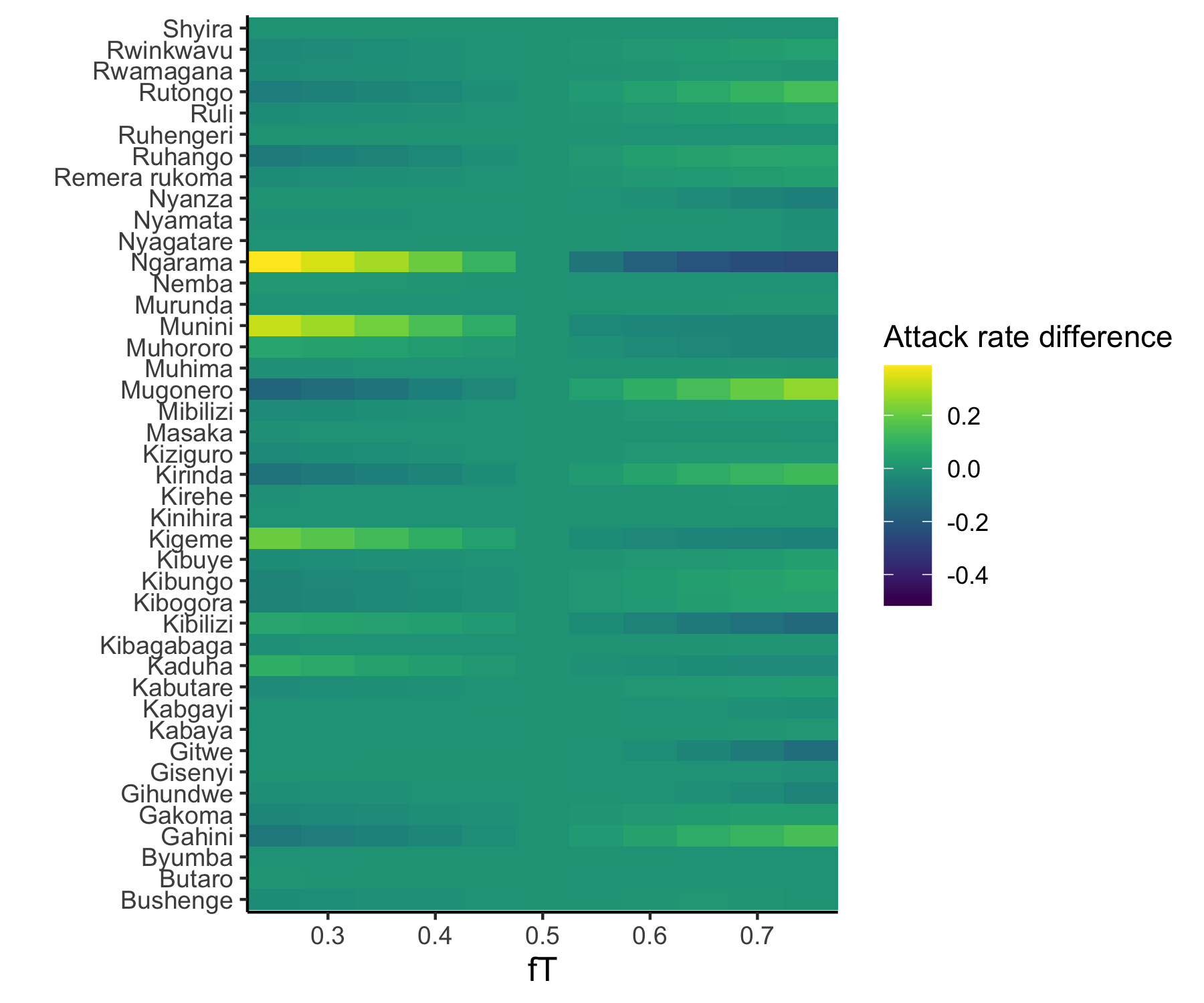


**Fig J in S1 Text**. Boxplots of average province-level attack rates of malaria incidence predicted under continuous simulation. In panel a) the average weekly attack rates per year are determined using the predicted effective population size as the denominator for the at-risk population. In panel b), the denominator population is the recent total census population data. The boxplots are displayed in order of increasing average attack rate in each panel. Jitter dots represent the average weekly attack rates for each study year.


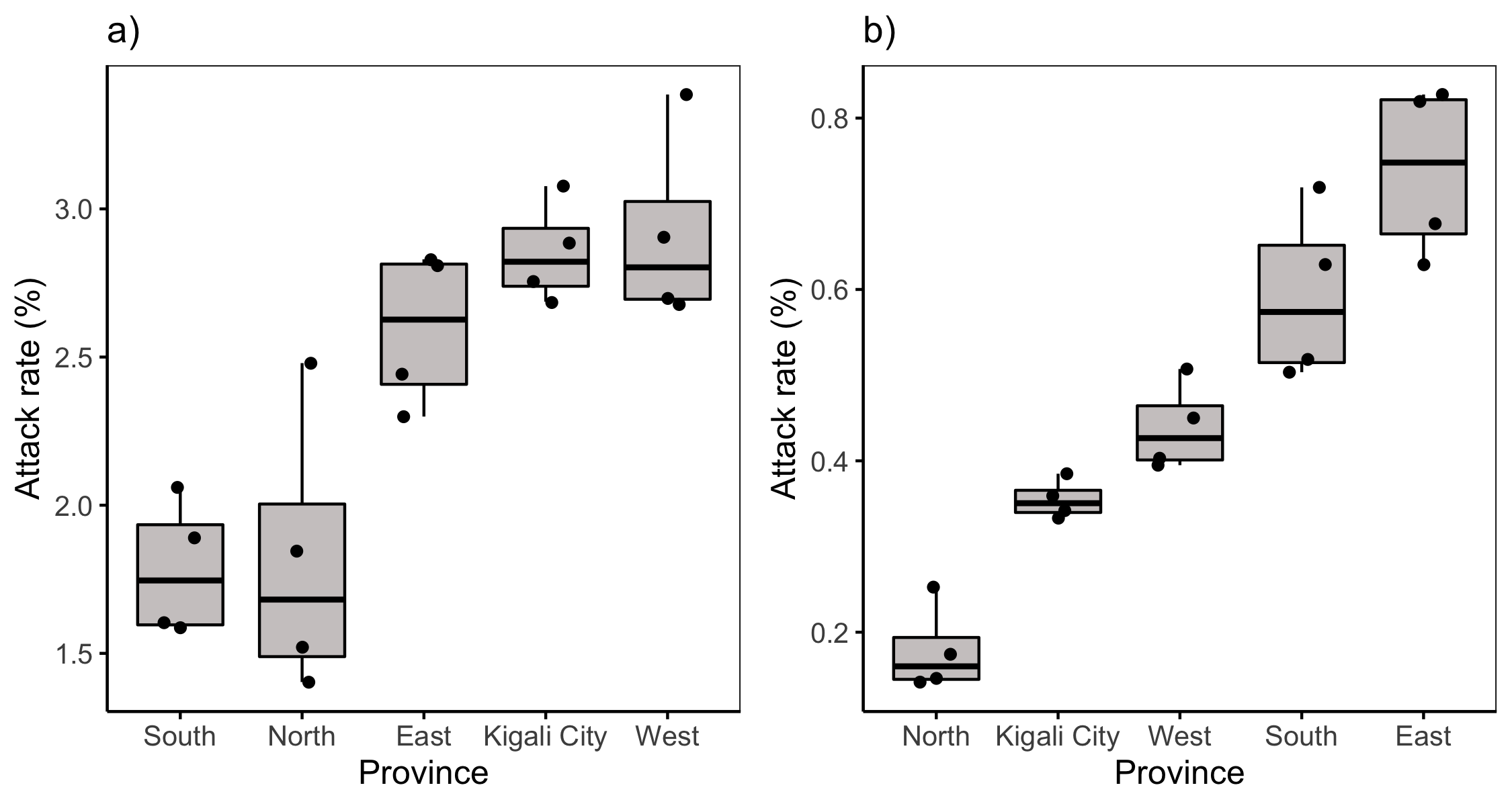


**Fig K in S1 Text**. The relationship between the malaria force of infection and model estimated weekly average EIR for sites found in the study provinces. The black solid line represents the theoretical, nonlinear relationship expected between the EIR and the force of infection as EIR increases. The open circles indicate the modeled force of infection for sites across the various provinces in Rwanda.


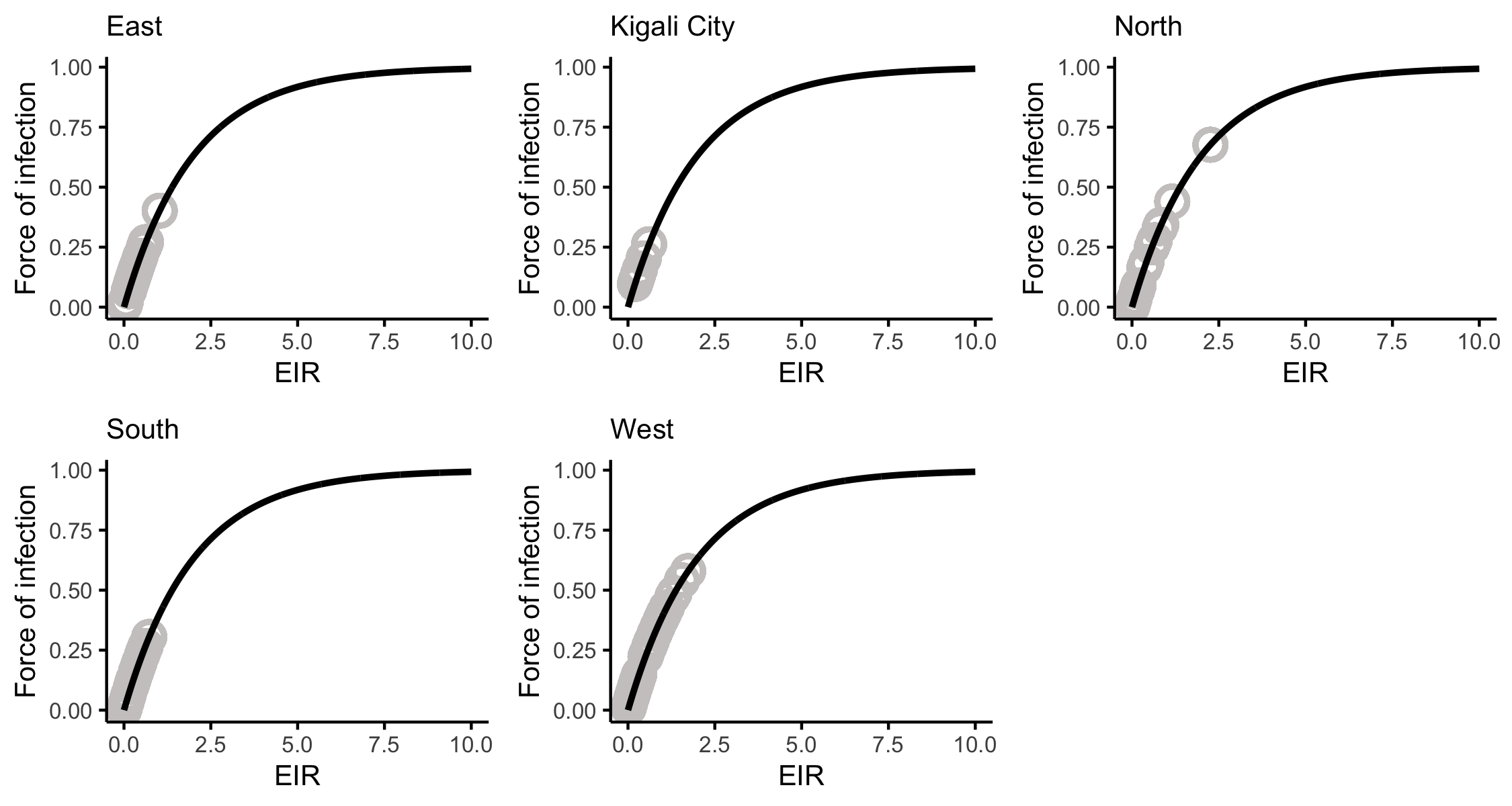


**References**

1. Piegorsch WW. Maximum likelihood estimation for the negative binomial dispersion parameter. Biometrics. 1990;46:863–7.

2. R Core Team. R: A language and environment for statistical computing. [Internet]. Vienna, Austria.: R Foundation for Statistical Computing.; 2019. Available from: https://www.R-project.org/.

3. National Institute of Statistics of Rwanda. Rwanda Demographic and Health Survey 2010. NISR, MOH, ICF International, Calverton, Maryland, USA; 2012.

4. National Institute of Statistics of Rwanda. Rwanda Demographic and Health Survey 2014-15. National Institute of Statistics of Rwanda (NISR) [Rwanda]; 2015.

5. Karema C, Wen S, Sidibe A, Smith JL, Gosling R, Hakizimana E, et al. History of malaria control in Rwanda: implications for future elimination in Rwanda and other malaria-endemic countries. Malar J [Internet]. 2020 [cited 2021 May 18];19. Available from: https://www.ncbi.nlm.nih.gov/pmc/articles/PMC7539391/

6. Laneri K, Bhadra A, Ionides EL, Bouma M, Dhiman RC, Yadav RS, et al. Forcing Versus Feedback: Epidemic Malaria and Monsoon Rains in Northwest India. PLOS Comput Biol. Public Library of Science; 2010;6:e1000898.

7. Yé Y, Hoshen M, Kyobutungi C, Louis VR, Sauerborn R. Local scale prediction of Plasmodium falciparum malaria transmission in an endemic region using temperature and rainfall. Glob Health Action. 2009;2.

8. McCann RS, Messina JP, MacFarlane DW, Bayoh MN, Vulule JM, Gimnig JE, et al. Modeling larval malaria vector habitat locations using landscape features and cumulative precipitation measures. Int J Health Geogr. 2014;13:17.

9. Lindsay SW, Parson L, Thomas CJ. Mapping the ranges and relative abundance of the two principal African malaria vectors, Anopheles gambiae sensu stricto and An. arabiensis, using climate data. Proc R Soc B Biol Sci. 1998;265:847–54.

10. Shililu JI, Grueber WB, Mbogo CM, Githure JI, Riddiford LM, Beier JC. Development and survival of Anopheles gambiae eggs in drying soil: influence of the rate of drying, egg age, and soil type. J Am Mosq Control Assoc. 2004;20:243–7.

11. Paaijmans KP, Imbahale SS, Thomas MB, Takken W. Relevant microclimate for determining the development rate of malaria mosquitoes and possible implications of climate change. Malar J. 2010;9:196.

12. Jacobs AFG, Heusinkveld BG, Nieveen JP. Temperature Behavior of a Natural Shallow Water Body during a Summer Period. Theor Appl Climatol. 1998;59:121–7.

13. Funk C, Peterson P, Peterson S, Shukla S, Davenport F, Michaelsen J, et al. A High-Resolution 1983–2016 Tmax Climate Data Record Based on Infrared Temperatures and Stations by the Climate Hazard Center. J Clim. American Meteorological Society; 2019;32:5639–58.

14. Funk C, Verdin A, Michaelsen J, Peterson P, Pedreros D, Husak G. A global satellite-assisted precipitation climatology. Earth Syst Sci Data. Copernicus GmbH; 2015;7:275–87.
